# Propelling consumer engagement *via* entrepreneurs' live streaming?

**DOI:** 10.3389/fpsyg.2022.890707

**Published:** 2022-08-03

**Authors:** Zheng Jiang, Haizhong Wang, Jiaolong Xue, Tianqi Zhai

**Affiliations:** ^1^School of Business, Sun Yet-sen University, Guangzhou, China; ^2^Business School, Sichuan University, Chengdu, China; ^3^Faculty of Business Administration, University of Macau, Macao, Macao SAR, China

**Keywords:** entrepreneurs' live streaming, reputation, guarantee, authenticity, admiration, consumer engagement

## Abstract

Entrepreneurs' live streaming (ELS) is an important tool for marketing, and it can increase consumer engagement, especially during the COVID-19 pandemic. Previous live streaming literature mainly focused on third-party live streaming, targeted at professional streamers and online celebrities. This study aims to discuss the factors underlying consumer engagement in the ELS. Using a mixed method of a quasi-experiment and an online survey, we analyzed the impact of the ELS on consumer engagement and the factors that drive consumer engagement in the ELS in each of 231 samples. In the enterprises' live streaming, the ELS has a significantly higher influence on consumer engagement compared with the employees' live streaming. In the ELS, based on source credibility theory and signaling theory, this study concludes that factors of ELS's credibility consist of internal factors (reputation, expertise, and interactivity) and external factors (guarantee, authenticity, and money-saving). The authors demonstrate that both internal and external factors positively affect trust in activities. Trust in activities positively affects consumer engagement and mediates the effects of reputation, expertise, interactivity, guarantee, and authenticity on consumer engagement. Moreover, reputation and expertise positively improve consumers' admiration toward the entrepreneur streamer and in turn, positively increase consumer engagement. Interactivity and expertise shorten the psychological distance. Psychological distance negatively affects consumer engagement and only helps increase the positive effect of interactivity on consumer engagement. These findings have theoretical and practical implications for live streaming e-commerce.

## Introduction

With the outbreak of COVID-19, companies have suffered massive economic losses. To recover their business, many entrepreneurs utilized the novel marketing tool—live streaming to promote their products and brands in person as a live streamer, which not only can boost sales volume but also try to give a vote of confidence to their employees to cope with the difficult times. For example, Mingzhu Dong, Chairwoman of GREE, has attracted more than 100 million viewers and achieved 47,600 million RMB gross merchandise volume (GMV) in 2020; Jun Lei, the founder of Mi, has attracted more than 105.13 million viewers and achieved 398 million RMB GMV by holding just three live streaming activities. Please check Appendix A1 in Supplementary material for statistics of other ELS activities. What are the factors that drive high GMV and attract a large number of viewers to ELS? Why do viewers trust ELS activities? Although those issues are important for both academic research and practice, few investigations have been conducted on this phenomenon.

Previous live streaming literature was mostly dedicated to third-party live streaming, which was anchored by professional streamers (e.g., Jiaqi Li) or online celebrities (e.g., Xinba, Cherie) (Park and Lin, 2020; Guo et al., 2022). Professional streamers and online celebrities are the third-party who use their experience to recommend valuable products to consumers *via* live steaming. Unlike those streamers, entrepreneurs, on behalf of their companies (Mazzarol and Reboud, 2020), market products to consumers in live streaming directly, a type of enterprises' live streaming. There are two types of enterprises' live streaming: the ELS and the employees' live streaming. We utilized a quasi-experiment to check the effect of two types of enterprises' live streaming on consumer engagement and we found that the ELS has a significantly higher impact on consumer engagement compared with live streaming by employees. Thus, it is more important to analyze the factors behind the successful consumer engagement of the ELS.

**Figure 1:**
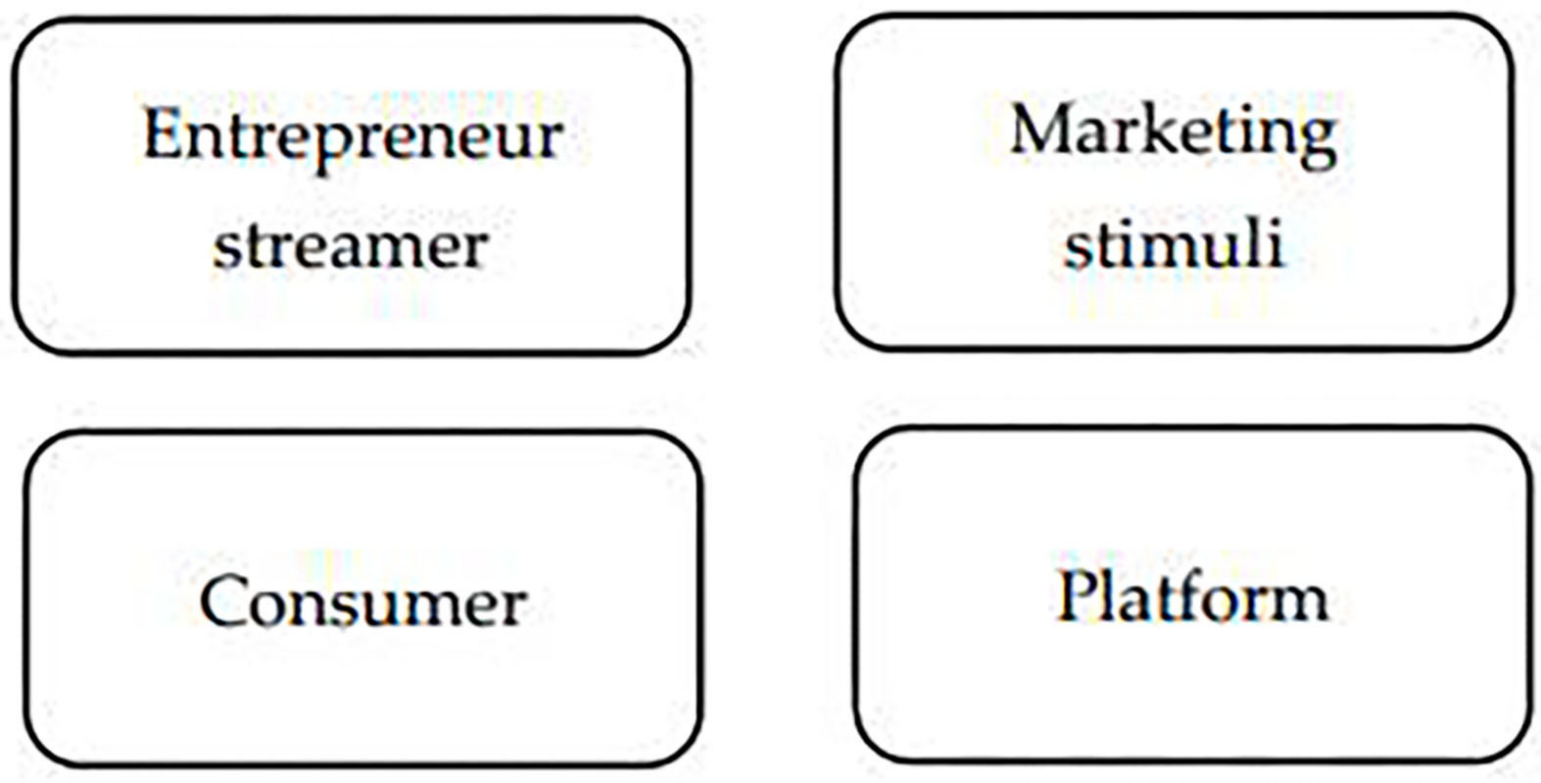
Components of the ELS.

Although previous studies have investigated consumer behavior in live streaming motivated by platform factors, such as IT affordance (Sun et al., 2019); by interrelationships in live streaming, such as trust-building (Guo et al., 2021), interpersonal factors (Chen et al., 2021); and by consumer perception factors, such as perceived value (Wongkitrungrueng and Assarut, 2020), online celebrities' credibility (Park and Lin, 2020), and most welcomed celebrities' characteristics (Guo et al., 2022). However, little attention has been given to the ELS and the driving factors of consumer engagement in the ELS. In the context of e-commerce, persons, products, and places are three key factors driving the effectiveness of live streaming e-commerce (iResearch, 2020). Previous studies have examined the effect of persons (streamers, consumers, and the relationship between consumers and streamers), products (congruence between streamers and products), and places (IT affordance of platforms) on the effectiveness of live streaming activities (Sun et al., 2019; Park and Lin, 2020; Guo et al., 2021). However, little attention has been shed on the effect of marketing stimuli. Marketing stimuli refer to the information provided by streamers in live streaming activities. We investigated the ELS activities in Taobao and Tik Tok, which are live streaming e-commerce platforms with billions of consumers, finding that entrepreneur streamers (sellers) and consumers (as buyers or viewers) are major persons. Since products provided by the ELS are produced or designed by their own company. It is more important for the entrepreneur streamer to provide effective marketing stimuli about the product in the ELS activities. Thus, we concluded that the ELS consists of four major components: entrepreneur streamers, consumers, marketing stimuli, and platform (see Figure 1).

Studies on source credibility have examined the credibility of the celebrity (Halder et al., 2021), the online celebrity (Liu et al., 2020; Meng et al., 2020), and the food online to offline (O2O) platform (Kang and Namkung, 2019). However, no study has examined the credibility of the ELS and its effect on consumer engagement. In the ELS, apart from entrepreneur streamers, the marketing stimuli provided in the ELS can greatly affect consumer behavior. According to signaling theory, the sender (an individual or an organization) can decide how to exchange signals (information) with the receiver (Spence, 1973; Connelly et al., 2011; Rokonuzzaman et al., 2021). Entrepreneur streamers can utilize the marketing stimuli to indicate the credibility of the ELS and then increase consumer engagement. Based on the philosophy behind the internal and external stimuli for online shopping (Kimiagari and Malafe, 2021), we propose the characteristics of entrepreneur streamers as internal factors and the marketing stimuli provided by entrepreneur streamers as external factors of the ELS's credibility. Moreover, consumer engagement refers to consumer behavioral performance toward entrepreneur streamers, including purchase behavior and non-transaction behavior (e.g., sharing, like, and rewarding) (Guo et al., 2021), which is important in live streaming e-commerce, but it was not examined in the ELS. Thus, based on source credibility theory and signal theory, this study aims to examine the effects of factors of the ELS's credibility on consumer engagement and the underlying mechanisms. We follow the logic of the S-O-R model to connect the relationships of independent variables, mediation variables, and dependent variables. The S-O-R model illustrates that environmental stimulus (S) impacts consumers' internal state (O), and in turn affects their responses (R) toward the subject in the online shopping context, which is extensively employed to explore the effect of environmental stimuli on consumer responses (Liu et al., 2013, 2016; Gao and Bai, 2014; Xue et al., 2020). In this study, internal factors and external factors of the ELS credibility (internal factors: reputation, interactivity, and expertise; external factors: guarantee, authenticity, and money-saving) are stimuli variables; admiration, psychological distance, and trust in activities are organism variables; consumer engagement is response variable.

Our findings provide four implications. First, this study extends the source credibility theory in the live streaming literature by providing the credibility of the ELS based on the source credibility theory and signaling theory. Second, this study provides a novel perspective on ELS-driven consumer engagement from the perspective of internal and external factors of the ELS's credibility, which enriches the literature on live streaming factors driving consumer engagement. Third, we examine the mechanism behind the ELS's credibility on consumer engagement, which helps us have a comprehensive understanding of consumer engagement in the ELS. Forth, this study adopted a method portfolio to discuss which factors and how the factors of the ELS affect consumer engagement through a quasi-experiment and online survey, hoping to provide a new way for scholars to study live streaming.

The rest of this paper is organized as follows. Section Literature review provides a literature review and presents the research hypothesis. Section Model development and assumption describes the empirical methodology used in this study. Section Research methods provides the results of the empirical design. Section Results discusses the major findings, theoretical and practical insights, and limitations.

## Literature review

### Source credibility theory and signaling theory

Source credibility theory refers “a source is perceived as possessing expertise relevant to the communication topic and can be trusted to give an objective opinion on the subject” (Ohanian, 1990). Previous research has widely accepted that source credibility consists of three dimensions, namely trustworthiness, expertise, and attractiveness (Ohanian, 1990). However, as the realities of consumption emerge, perhaps some other new elements are relevant to the components of source credibility. Ismagilova et al. (2020); Halder et al. (2021) suggested that celebrity credibility needs multiple dimensions, rather than just trustworthiness, expertise, and attractiveness. In live streaming consumption, Meng et al. (2020) introduced that interaction, as one of the online celebrity factors of streamers' credibility, has a positive impact on purchase intention. In the food O2O consumption context, Kang and Namkung (2019) considered reputation as one of the source credibilities of the O2O platform in customers' evaluation. Besides, according to signaling theory, the sender can manipulate the information to influence decisions in the exchange relationship and to indicate quality and trustworthiness (Wagner et al., 2011; Rokonuzzaman et al., 2021). Rokonuzzaman et al. (2021) studied how retailers' reopening policies can reduce perceived risk and improve store image and retain patronage intention. In this study, based on signaling theory, we propose that the marketing stimuli (guarantee, authenticity, and money-saving) provided in the ELS can serve as the signal for consumers to indicate the credibility of the ELS. In the online shopping context, studies have classified internal and external stimuli affecting online consumption (Lim and Yazdanifard, 2015). Studies have classified individual characteristics as internal factors (Chan et al., 2017) and classified marketing stimuli as one of the external factors (Kimiagari and Malafe, 2021). In the ELS, unlike other streamers, entrepreneur streamers can not only showcase their products but also decide the marketing stimuli in the live streaming. Based on the philosophy behind the internal and external stimuli of online shopping (Kimiagari and Malafe, 2021), we propose that the characteristics of entrepreneur streamers (reputation, expertise, and interactivity) as internal factors and the marketing stimuli (guarantee, authenticity, and money-saving) provided in the ELS as external factors of the ELS's credibility.

### Live streaming e-commerce-related research

Previous literature on live stream E-commerce mainly focused on online celebrities (Liu et al., 2020; Park and Lin, 2020) and professional streamers (Addo et al., 2021; Chen et al., 2021). Studies on online celebrities have mainly focused on (1) trustworthiness, attractiveness, and live streaming content (Wanghong vs. self)-product fit (Park and Lin, 2020), (2) online celebrities' image and product consistency (Liu et al., 2020), (3) celebrity characteristics (i.e., credibility, professionalism, skill, interaction, and attraction) (Meng et al., 2020) on purchase intention. Studies on professional streamers have mainly dedicated to (1) user stickiness affected by technical factors (synchrony and alternative expressions) and social factors (interaction and identification); (2) purchase intention affected by social capital and negative event (Xu et al., 2022); consumer engagement (Addo et al., 2021), interpersonal factors (perceived expertise, perceived similarity, perceived familiarity and perceived likeability) (Chen et al., 2021), crosss-border E-commerce live streaming features (Guo et al., 2021); social status (Hou et al., 2020), IT affordability (Sun et al., 2019); (3) intention to watch influenced by interactivity and top streamers' personal traits (Guo et al., 2022); sense of community, interactivity, and emotional support (Chen and Liao, 2022); (4) tipping and gift-giving intention affected by audience (Lu et al., 2021), live streamers' personal traits (trustworthiness, expertise, attractiveness) and live scene characteristics (telepresence, instant feedback, interactivity, entertainment) (Li and Peng, 2021); (5) consumer engagement influenced by interactivity (Kang et al., 2021), trust (trust in community members, trust in broadcasters, trust in products) (Guo et al., 2021), streamers' emotion (Lin et al., 2021), perceived values (Wongkitrungrueng and Assarut, 2020), financial bonds, and social bonds structural ties (Hu and Chaudhry, 2020), and live interaction (Xue et al., 2020). A recent study on celebrity streamers has investigated that interactivity has a positive effect on purchase intention through social presence and flow experience. Related studies are given in Table 1.

**Table 1:** Related live streaming empirical research.

**No**.	**References**	**Method**	**Live streamer type**	**Theory**	**IV**	**MoV**	**MeV**	**DV**	**Key findings**
1	Guo et al. (2022)	Online survey	Professional streamer	Social judgment and interpersonal communication	Streamer characteristics (beauty, warmth, expertise, humor, and passion)	Utilitarian value, hedonic value		Popularity, watching intention, purchase intention	Streamers characteristic affects streamer's popularity, consumer's watching intention and purchase intention *via* perceived values.
2	Xu et al. (2022)	Survey	Professional streamer	Information asymmetry theory, parasocial relationship theory	Social capital, negative event	Parasocial relationship	Trust	Purchase intention	Professionalism, reciprocal and parasocial, relationship positively affect purchase intention. Negative public events negatively affect consumers' purchase intention. The scale of live streaming and the streamer's commitment insignificantly affect purchase intention. Trust meditated the effect of professionalism and parasocial relationship on purchase intention.
3	Chen and Liao (2022)	Survey	Professional streamer	Social presence theory	Sense of community, interactivity, and emotional support	Streamer attractiveness	Social presence	Live streaming watching	Sense of community, interactivity, and emotional support have positive effect on social presence, in turn leading to consumers' watching. Streamer attractiveness moderates the effect of social presence and streaming watching.
4	Addo et al. (2021)	Secondary data	Professional streamer		Customer engagement	Price	Followship	Purchase intention	Customer engagement has an association with followship and purchase intention. Additionally, price is the moderator of the effect of customer engagement on followership and purchase intention.
5	Guo et al. (2021)	Survey	Professional streamer	Trust transfer theory	Trust in community members, trust in broadcasters, trust in products		Swift guanxi	Customer engagement	Trust in community members, broadcasters, and products have a positive effect on customer engagement through swift guanxi.
6	Kang et al. (2021)	Real-time data	Professional streamer	Stimulus–organism–response	Interactivity		Tie strength	Customer engagement	This study finds that interactivity has a dynamic effect on customer engagement, which is mediated by tie strength in live streaming commerce.
7	Lu et al. (2021)	Field experiment	Professional streamer	Social image concerns, Herding	Audience size			Pay-what-you-want	The results reveal that audience size has a concave effect on pay-what-you-want. Specifically, the effect of audience size becomes negative when audience size is relatively large.
8	Li et al. (2021)	Survey	Professional streamer	Attachment theory, socio-technical approach	Interaction, identification, synchronicity, vicarious expression		Emotional attachment to streamers, platform attachment	Visit duration, user retention	Technical factors (synchronicity and vicarious expression) and social factors (interaction and identification) have a positive effect on emotional attachment to streamers and platform attachment, respectively, which in turn prompt user stickiness.
9	Chen et al. (2021)	Survey	Professional streamer	Stimulus–organism–response	Interpersonal factors		Swift guanxi	Purchase intention	Interpersonal factors (i.e., perceived expertise, perceived similarity, perceived likeability, and perceived likeability) affect purchase intention, which is mediated by swift guanxi.
10	Lin et al. (2021)	Secondary data	Professional streamer		Broadcaster emotion			Audience emotion, viewers' engagement activities	Broadcasters with happier emotions make the audience happier and attract viewers' engagement activities.
11	Guo et al. (2021)	Survey	Professional streamer	S-O-R theory	Crosss-border E-commerce live streaming features	Saving money	Overall perceived value, overall perceived uncertainty	Cross-border purchase intention	Live streaming features positively affect overall perceived value and purchase intention, and negatively affects overall perceived uncertainty; moreover, saving money enhances the effect of live streaming features on overall perceived value.
12	Sun et al. (2021)	Survey	Celebrity streamer	S-O-R theory	Interactivity		Social presence, flow experience	Purchase intention	Interactivity has a positive effect on purchase intention through social presence and flow experience.
13	Xue et al. (2020)	Survey	Professional streamer	Stimulus–organism–response	Live interaction	Susceptibility to informative influence	Perceived usefulness, perceived risk, psychological distance	Social commerce engagement	Live interactions (i.e., personalization, responsiveness, entertainment, mutuality, perceived control) have a positive effect on perceived usefulness and harm perceived risk and psychological distance, facilitating social commerce engagement.
14	Park and Lin (2020)	Survey	Internet Celebrity (Wanghong)	Source credibility, match-up hypothesis	Wanghong-product fit, live content-product fit, self-product fit		Wanghong trustworthiness, Wanghong attractiveness, Utilitarian attitude, Hedonic attitude	Intention to buy	Wanghong-product fit positively affects Wanghong attractiveness and Wanghong trustworthiness, while live content-product fit positively affects utilitarian and hedonic attitudes toward the content. Source trustworthiness, hedonic attitude, and self-product fit positively affect the intention to buy.
15	Hu and Chaudhry (2020)	Survey	Professional streamer	Stimulus–organism–response	Relational bonds		Affective commitment to the broadcaster, affective commitment to the online marketplace	Consumer engagement	Social and structural bonds have positive direct effects and an indirect effect *via* affective commitment on consumer engagement, while financial bonds only indirectly affect consumer engagement through affective commitment.
16	Liu et al. (2020)	Interview, big data, survey	Internet celebrity (Wanghong)	Parasocial interaction, Flow theory	Credibility, professionalism, interactivity, attractiveness	Wanghong' image and product consistency	Perceived hedonic shopping value, perceived practical shopping value	Purchase intention	Credibility, professionalism, interactivity, and attractiveness influence perceived hedonic shopping value and perceived practical shopping value, promoting purchase intention. Wanghong's image and product consistency moderate the influences.
17	Wongkitrungrueng and Assarut (2020)	Survey	Professional streamer		Perceive values		Trust in products, trust in sellers	Customer engagement	Symbolic value directly and indirectly *via* trust in sellers affects customer engagement, while utilitarian and hedonic values indirectly through customer trust in products and trust in sellers sequentially affect customer engagement.
18	Hou et al. (2020)	Interview, survey	Professional streamer	Uses and gratification theory, social interaction, entertainment	Interactivity, social status display, humor appeal, Sex appeal			Continuing watching intention, consumption intention	Sex and social status influence consumption intention, while interactivity and humor appeal influence continuous watching intention. The effects of these factors vary across different types of live streaming (i.e., event, education, and personal sharing).
19	Sun et al. (2019)	Survey	Professional streamer	IT affordance	Visibility, metavocing, guidance shopping		Live streaming shopping engagement	Customer behavior (purchase intention)	IT affordance (i.e., visibility affordance, metavoicing affordance, and guidance shopping affordance) positively affects customer purchase intention *via* live streaming engagement.

IV, Independent variable; MoV, Moderating variable; MeV, Mediating variable; DV, Dependent variable.

The existing literature related to live streaming is increasingly focused on consumer engagement. Consumer engagement is an important factor in predicting and explaining consumer repurchase intention and preference for brands (Xue et al., 2020). With the emphasis on the importance of engagement in social commerce, engagement has transpired in live E-commerce recently (Guo et al., 2021; Kang et al., 2021). In the live streaming context, according to Guo et al. (2021), we define consumer engagement as consumers' behavioral engagement toward entrepreneur streamers, including purchase behavior and non-transaction behavior (e.g., sharing, like, and giving tips). Live streaming literature on consumer engagement mostly focused on live streaming activities hosted by professional streamers from third-party companies (e.g., MCN) and overlooked the live streaming activities hosed by the enterprises themselves. There are two types of live streaming activities hosted by the enterprises themselves: ELS and employees' live streaming. In this study, we aim to figure out the factors behind the ELS-driven consumer engagement, which is significantly higher than employees' live streaming.

## Model development and assumption

### The effect of reputation on admiration and trust in activities

#### The effect of reputation on admiration

According to Ferris et al. (2003), entrepreneurial reputation can be defined as a perceptual identity that reflects the complex combination of the entrepreneur streamers' traits and accomplishments, demonstrated behaviors, and expected images directly observed and/or reported from secondary sources over a period of time. Haidt (2003) provided that preference originates from an appreciation of excellent behavior and characters. A previous study has examined that the entrepreneurs' image positively affects consumers' admiration for the entrepreneurs (Liu et al., 2018). Entrepreneurs' image contains two important sub-categories: competence and charisma image (Park and Berger, 2004). Likewise, we define that entrepreneur reputation contains competence reputation and charisma reputation. Entrepreneur reputation represents that the entrepreneur streamers have competence and charisma image for a long time, which leads consumers to appreciate the behavior and unique character of the entrepreneur streamers. Thus, we propose that:

**H1a:** Reputation is positively associated with admiration for entrepreneur streamers.

#### The effect of reputation on trust in activities

Ranft et al. (2006) illustrated that people tend to trust an individual with an established reputation and trust that the person will behave as usual. In e-commerce, sellers' reputations can reduce consumers' information asymmetry and enhance their acceptance of e-commercial activities (Grossman and Stiglitz, 1980; Ruohomaa and Kutvonen, 2005). Kim and Peterson (2017) illustrated that reputation is an important prerequisite for trust. In the ELS, we assume that entrepreneur reputation prompts trust toward live broadcast activity.

**H1b:** Reputation is positively associated with trust in activities.

### The effect of expertise on admiration, psychological distance, and trust in activities

#### The effect of expertise on admiration

Expertise is one of the source credibility dimensions and is defined as “one's skills and competence in delivering genuine and accurate information” (Ohanian, 1990). Previous studies have suggested that admiration is derived from excellent behaviors, such as skills, achievements, and actions (Haidt, 2003; Algoe and Haidt, 2009). In the ELS, entrepreneur streamers are familiar with their products and know the story of the products. In the live streaming activities, entrepreneur streamers showcase their expertise in products and promotion skills, which will boost consumers' admiration. Thus, we propose that:

**H2a:** Expertise is positively associated with admiration for entrepreneur streamers.

#### The effect of expertise on psychological distance

The expertise of entrepreneur streamers may help consumers reduce uncertainty and shorten the psychological distance. Previous studies have suggested that source expertise has a role in persuasion and in enhancing brand recognition, which then increases purchase intention (Ohanian, 1990; Ladhari et al., 2020). Chen et al. (2021) suggested that perceived expertise affects interpersonal relationships (swift guanxi) in live streaming activity. In the ELS, expertise may close the psychological distance between consumers and entrepreneur streamers. Hence, we propose that:

**H2b:** Expertise is negatively associated with psychological distance.

#### The effect of expertise on trust in activities

In the live broadcast context, Chen et al. (2021) examined that perceived expertise has a positive effect on purchase intention through quickly established social ties. Liu et al. (2020) found that professionalism (expertise) has a positive effect on purchase intention in live broadcast activities anchored by online celebrities. Entrepreneur streamers have rich knowledge of products, and they are experts on their products and services. People trust experts and are more likely to adopt their opinions. In e-commerce platforms, service providers with professional skills and knowledge can provide useful information for their customers, thereby reducing information asymmetry (Dimoka et al., 2012). Moreover, consumers are inclined to have higher trust when the information provided by sellers is relevant and useful. Thus, we assume that the expertise of entrepreneur streamers will boost consumers' trust in live broadcast activity. Therefore, we propose that:

**H2c:** Expertise is positively associated with trust in activities.

### The effect of interactivity on psychological distance and trust in activities

#### The effect of interactivity on psychological distance

Interactivity undermines consumer engagement in live broadcast (Lim et al., 2012; Xue et al., 2020). In the ELS, entrepreneurs can not only promote their products and brands but also share their personal entrepreneurial experiences, so that consumers are provided with more opportunities to interact with entrepreneurs directly, which plays a crucial role in customers' attitudes. In the ELS, given the initial distance between entrepreneur streamers and consumers, we assume that interactivity can significantly shorten the psychological distance between consumers and entrepreneur streamers. Thus, we propose that:

**H3a**. Interactivity is negatively associated with psychological distance.

#### The effect of interactivity on trust in activities

In the live stream context, Xue et al. (2020) defined live broadcast interaction as “the degree to which streamers, consumers, or more communication parties can interact with each other in real time, on the communication media channels, and on the messages and the degree to which such influences are synchronized in live broadcast e-commerce.” Xue et al. (2020) have examined that interaction can reduce the perceived risk in live streaming. In view of the rare opportunity for consumers to communicate with entrepreneurs, we mainly focused on the interactivity between consumers and entrepreneur streamers. Studies on streamers' characteristics have illustrated that interactivity can enhance perceived value (Liu et al., 2020) and enhance purchase intention (Meng et al., 2020) in live broadcast activity. Interactivity has a positive effect on trust through the traffic in retailers' AR-apps (Arghashi and Yuksel, 2022). In the ELS, based on previous studies, we propose that:

**H3b:** Interactivity is positively associated with trust in activities.

### The effect of guarantee on trust in activities

Guarantee means that consumers have higher services/products evaluation (Ostrom and Iacobucci, 2016). Desmet (2014) noted that retailers can normally offer two types of guarantee: the manufacturer's quality guarantee required by law and the money-back guarantee. The former is provided by suppliers (repair, exchange, and refund due to the quality issue in a certain time) while the latter provides consumers a chance to return products purchased without any condition within a certain period of time. Professional streamers and online celebrities are the third parties to promote products, while entrepreneur streamers represent the manufacturers/brand owners, and their products are legally obligated to provide the guarantee from manufacturers. Once consumers have product quality issues in live broadcast, they need to seek support from product suppliers or manufacturers. Rokonuzzaman et al. (2021) illustrated that retailers' reopening policy of retailers enhances store image and retains patronage intention *via* decreasing perceived risk. Moreover, some entrepreneur streamers provide a money-back guarantee. For example, Jianzhang Liang, founder of Ctrip, promised that consumers can get a refund if they want to return the products (e.g., hotel booking and sightseeing tickets) in a year. The guarantee provided by entrepreneur streamers gives consumers faith in their products and the whole live broadcast activity. Thus, we propose that:

**H4:** Guarantee is positively associated with trust in activities.

### The effect of authenticity on trust in activities

Authenticity refers to “a multidimensional structure variously consisting of claims that an entity is consistent in its internal values and external behaviors; it conforms to relevant social norms, and the person, time, or place it claims to be” (Lehman et al., 2019). In the ELS, we defined authenticity as that consumers' perception of the products, information, and activity provided by the live streaming activity conforms to the information claimed by the entrepreneur streamers. Eggers et al. (2013) noted that brand authenticity is important for gaining trust. Moreover, Matthews et al. (2020) found that the frontline service employees' authenticity can enhance consumers' perceived trust, which in turn affects their purchase intention. Although live streaming activities have grown rapidly in recent years, some live streamers sell fake products for chasing high profits without market supervision. However, entrepreneur streamers who represent their organizations and brands will not deceive consumers in the live streaming activities that they can ensure the authenticity of the products, which will allow them to gain more trust from consumers. Thus, we propose that:

**H5:** Authenticity is positively associated with trust in activities.

### The effect of money-saving on trust in activities

Chandon et al. (2000) noted that due to the lower price, live streaming activities bring the impression of money-saving to consumers. Previous studies have examined that saving money is one of the vital factors for consumers to engage in their relationships and behaviors (Park et al., 2012; Hu and Chaudhry, 2020). In live streaming, discounts, gifts, and special prices can be offered by streamers to help customers save money (Hallanan, 2019; Hu and Chaudhry, 2020). However, in reality, consumers sometimes complain that they receive a false price discount or higher price compared with other marketing activities. Different from other streamers, entrepreneur streamers have the power to offer the “ex-factory price”—the lowest product price for consumers. It can make consumers save the largest amount of money, which is expected to greatly improve consumers' trust in live streaming activity. Thus, we propose that:

**H6:** Money-saving is positively associated with trust in activities.

### The effect of admiration on consumer engagement

Schindler et al. (2013) defined admiration as “respect for someone or something considered praiseworthy or excellent.” Admiration has an impact on consumer emotional and behavioral consequences (Aaker et al., 2012). In terms of admiration for something, existing research has examined the effect of brand admiration on purchase intention (Trivedi and Sama, 2020; Gupta et al., 2021). Gupta et al. (2021) examined the mediating role of brand admiration in the influence of CSR communication *via* social media on consumers' purchase intention. Trivedi and Sama (2020) have pointed out that preference for a brand has a positive effect on online purchase intentions. In terms of admiration for someone, some scholars have examined the positive effect of admiration for employees on engagement (He et al., 2019) and the positive effect of consumer admiration for entrepreneurs on brand attitude (Liu et al., 2018). In the ELS, we assume that consumer admiration for entrepreneur streamers has a positive influence on consumer engagement.

**H7:** Admiration is positively associated with consumer engagement.

### The effect of psychological distance on consumer engagement

The psychological distance can be defined as “the extent to which individuals mentally perceive the entrepreneur streamers as being distant from themselves at that moment” (Liberman and Trope, 2008). Xue et al. (2020) suggested that psychological distance negatively affects social commerce engagement in live streaming. Hernández-Ortega (2018) noted that shortening the social–psychological distance results in increased purchase intention. Additionally, Edwards et al. (2009) illustrated that the reduction of psychological distance will reduce individuals' sense of danger and crisis, barriers, and self-defense, and will provide real, genuine, more inclusive, and trusting feelings (Edwards et al., 2009). Thus, we propose that:

**H8:** Psychological distance is negatively associated with consumer engagement.

### The effect of trust in activities on consumer engagement

Trust has a positive effect on consumer engagement in live streaming (Wongkitrungrueng and Assarut, 2020; Guo et al., 2021). Wongkitrungrueng and Assarut (2020) examined that trust in sellers and products has a positive relationship with consumer engagement in live broadcasts. Extending the studies of Wongkitrungrueng and Assarut (2020), and Guo et al. (2021) have examined that trust in community members, trust in broadcasters, and trust in products have a positive effect on consumer engagement through quickly established social bonds. However, due to the negative news that some live streamers provide false information about products/services, consumers still have concerns about the credibility of live streaming activity (iResearch, 2020). In online/offline commerce, consumers trust the following several entities: the company, the agent (seller, salesperson, and website), the product, and the market/channel (Komiak and Benbasat, 2004). In the ELS, based on the ideas of Komiak and Benbasat (2004), we defined trust as trust toward the whole live streaming activity, including products, entrepreneur streamer, and information provided in live streaming. Friedrich et al. (2019) examined that trust positively affects website stickiness. We assume that consumers who have trust in live streaming activity will actively engage in the ELS.

**H9:** Trust in activities is positively associated with consumer engagement.

### The mediating role of admiration, psychological distance, and trust in activities

#### The mediating role of admiration

As mentioned before, appreciation may mediate the impact of reputation and expertise on consumer engagement. Consumers may engage in the live broadcast because of the admiration aroused by internal factors of the ELS's credibility (i.e., reputation and expertise). Thus, we propose that:

**H10a:** Admiration mediates the effect of reputation on consumer engagement.
**H10b:** Admiration mediates the effect of expertise on consumer engagement.

#### The mediating role of psychological distance

In the ELS, as analyzed before, the psychological distance may mediate the effects of interactivity and expertise on consumer engagement. Entrepreneur streamers with expertise can interact with consumers in real time and showcase products in person, which can significantly narrow the psychological distance between the entrepreneurs and consumers in e-commercial live broadcasts, which in turn prompts consumer engagement. Thus, we propose that:

**H11a:** Psychological distance mediates the effect of interactivity on consumer engagement.
**H11b:** Psychological distance mediates the effect of expertise on consumer engagement.

#### The mediating role of trust in activities

As mentioned earlier, entrepreneurs' reputation, expertise, interactivity, guarantee, authenticity, and money-saving have a positive influence on trust, and trust has a positive influence on consumer engagement. Thus, we propose that:

**H12a–f:** Trust in activities mediates the relationship between (a) reputation; (b) expertise; (c) interactivity; (d) guarantee; (e) authenticity; and (f) money-saving and consumer engagement.

**Figure 2:**
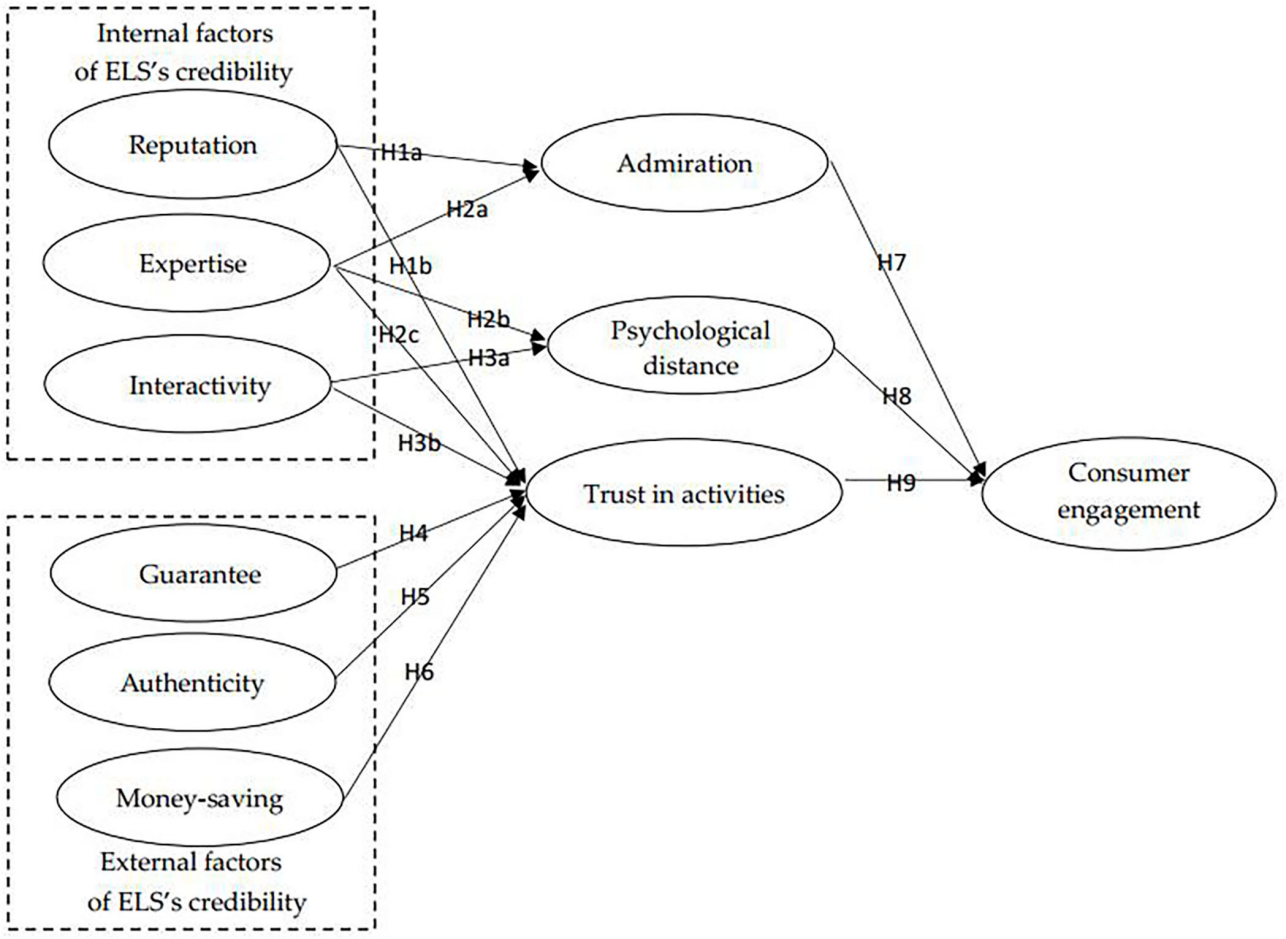
Research model.

The overview of these relationships in this research model is shown in Figure 2.

## Research methods

### Study 1: Quasi-experiment

We first conducted a quasi-experiment to examine the effect of entrepreneur live streaming on consumer engagement. The quasi-experimental approach was a good approximation of a randomized experiment, especially when a randomized experiment is not available. We collected our data from Tik Tok, which is one of the biggest live streaming and short video platforms in China. According to the Annual Data Report of 2020 (Tik Tok, 2021), the amount of daily active users of Tik Tok was more than 6 billion at the end of August 2020. The data contain details of the live streaming activity anchored by the entrepreneur streamers on 1st January and the live broadcast activity anchored by the employee streamers on 5th January from the Tik Tok platform. Each live streaming activity was operated from 20:00 to 22:00. For the consumer engagement, we collected the number of viewers in the peak, the total amount of viewers, the number of viewers' likes, the total amount of rewards, and the increased number of fans every 2 min.

We used an independent sample method, *t*-test to examine differences in consumer engagement between the entrepreneur streamers and the employee streamers. The results showed that consumer engagement driven by the entrepreneur streamers is higher than the employee streamers. In detail, the peak amount of viewers in the entrepreneur live streaming [*M*_EPS_ = 505539.15] is significantly higher than that of the employee live streaming [*M*_EMS_ = 7144.91, *t*_(53)_ = 29.198, *p* < 0.001]; the total amount of viewers in the entrepreneur live streaming (*M*_EPS_ = 12472506.83) is significantly higher than that of the employee live streaming [*M*_EMS_ = 1460674.81, *t*_(53)_ = 12.344, *p* < 0.001]; the number of viewer's likes in the entrepreneur live streaming [*M*_EPS_ = 17267900.89] is significantly higher than that of the employee live streaming [*M*_EMS_ = 325758.96, *t*_(53)_ = 20.994, *p* < 0.001]; the total amount of tips in the entrepreneur live streaming (*M*_EPS_ = 1452402.54) is significantly higher than that of the employee live streaming [*M*_EMS_ = 13183.83, *t*_(53)_ = −32.056, *p* < 0.001]; the increased number of fans in the entrepreneur live streaming (*M*_EPS_ = 302424.54) is significantly higher than that of the employee live streaming [*M*_EMS_ = 28068.87, *t*_(53)_ = 11.871, *p* < 0.001]. Descriptive statistics are shown in Table 2.

**Table 2:** Descriptive statistics.

		**Frequency**	**Mean**	**SD**	**MIV**	**MAV**
The peak amount of viewers	EPS	54	505,539.15	125,410.159	200,422	743,058
	EMS	54	7,144.91	2,510.461	4,721	15,219
The total amount of viewers	EPS	54	12,472,506.83	6,551,439.668	2,181,319	24,017,339
	EMS	54	1,460,674.81	221,789.535	139,780	1,711,887
Number of viewer's likes	EPS	54	17,267,900.89	5,930,260.260	4,947,264	26,061,681
	EMS	54	325,758.96	18,660.953	290,277	352,197
The total amount of tips	EPS	54	1,452,402.54	407,367.564	113,064	1,872,094
	EMS	54	13,183.83	820.160	11,336	14,480
The increased number of fans	EPS	54	302,424.54	169,817.986	54,962	589,440
	EMS	54	28,068.87	1,895.021	24,105	30,593

EPS, entrepreneur streamer; EMS, employee streamer; SD, standard deviation; MIV, minimum value; MAV, maximum value.

Study 1 shows that ELS has a more significant impact on consumer engagement in corporate live streaming environments than employees' live streaming. The next study aims to uncover the success factors behind consumer engagement and the mechanisms in the ELS.

### Study 2: Online survey

#### Measurement scales

To measure the 10 constructs in this study, we adopted items based on existing literature. We modified the questionnaires slightly to reflect the context of the ELS: admiration, adapted from Algoe and Haidt (2009) and Schindler et al. (2013); authenticity, adapted from Schaefer and Pettijohn (2006); Kim and Lee (2019b); reputation, adapted from Ferris et al. (2003) and Park and Berger (2004); expertise, adapted from Ohanian (1990) and Liu et al. (2020); consumer engagement, adapted from Hollebeek et al. (2014) and Guo et al. (2021); guarantee, adapted from Desmet (2014); interactivity, adapted from Kim and Lee (2019a) and Xue et al. (2020); money-saving, adapted from Chandon et al. (2000) and Hu and Chaudhry (2020); psychological distance, adapted from Liberman and Trope (2008) and Xue et al. (2020); and trust in activities, adapted from Komiak and Benbasat (2004) and Kim and Park (2013). See Appendix A2 in Supplementary material for definitions and Appendix A3 in Supplementary material for measurement scales.

#### Sample and data collection

We distributed the questionnaire on the Wenjuanxing website (https://www.wjx.cn/), one of the largest professional data collection websites in China, which has collected more than 13.14 billion questionnaires. Consistent with the sample methods employed by Gao et al. (2021) and Guo et al. (2021), we utilized Wenjuanxing.com as our sampling platform. We choose to use the sample service offered by Wenjuanxing, a professional data collection institution for research purposes. Wenjuanxing helped us to randomly select ELS watchers and to remove invalid questionnaire responses that failed attention check items, such as the repetition and reverse questions to remove invalid survey. We employed an inverse method to ensure the translation accuracy of the survey. Moreover, senior and leading scholars studying e-commerce were invited to examine the applicability of the survey for the live streaming context. Before the official online survey, we conducted pilot studies to assess the reliability and validity of the scales.

At the very beginning of the questionnaire, we set the question “Have you watched the entrepreneurs' live streaming?” to screen those who have watched the entrepreneur live streaming to proceed on the questionnaire. In addition, we also provide a video clip about ELS activities for participants to arouse their memory about ELS activities.

A total of 300 samples were obtained. All the variables were measured on a 7-point Likert-type scale from strongly disagree (1) to strongly agree (7). After omitting invalid questionnaires because of logic inconsistency, a total of 231 samples were available for data analysis. The sample size was ≈25 times the number of constructs and the effective sampling rate was 77%. The sample demographics are shown in Table 3.

**Table 3:** Sample demographics (*N* = 231).

**Variable**	**Category**	**Number**	**Percentage (%)**
Gender	Male	95	41.1
	Female	136	58.9
Age	≤18	5	2.2
	18–25	59	25.5
	26–30	56	24.2
	31–40	94	40.7
	41–50	13	5.6
	51–60	4	1.7
	≥60	0	0
Education	Middle school or below	1	0.4
	High school	2	0.9
	Junior college	20	8.7
	Bachelor's degree	187	81.0
	Master's degree or higher	21	9.1
Monthly disposable income (RMB)	<1,000	14	6.1
	1,000–3,000	31	13.4
	3,001–5,000	38	16.5
	5,001–7,000	58	25.1
	>7,000	90	39.0
Times of purchasing by watching live streaming or sharing shopping information about live streaming (past 3 months)	1–2	47	20.3
	3–4	71	30.7
	5–6	60	26.0
	≥7	53	22.9

## Results

Partial least squares structural equation modeling (PLS-SEM) was employed in our study. PLS-SEM is an analytical method with good performance in validation and predictive assessment capability (Fornell and Bookstein, 1982). PLS-SEM can be used regardless of whether the data are normally distributed or not. In addition, PLS-SEM is superior to surveys with sample sizes of fewer than 500 (Hair et al., 2014). In this study, we performed PLS-SEM analysis with SmartPLS 3.2.8. First, we analyzed the reliability and validity of the measures by assessing the measurement model. After that, we tested the proposed hypotheses through a structured model. To ensure the research validity, we discuss common method bias in the following part.

### Common method bias

Since we collected data *via* questionnaire, common method bias (CMB) could be a potential problem. First, we used Harman's single-factor test to check the method variance in the data. The results show that 10 factors with eigenvalues >1.0 are formed. The largest factor accounted for 29.879%, below 50% of the variance of subjects indicating that CMB did not pose a threat in our study (Fornell and Bookstein, 1982). Second, we built a technique for fine-grained CMB testing using unmeasured latent methods (Podsakoff et al., 2003). The results show that the average variance explained by the substantive structure (0.568) is much higher than that explained by the common method factor (0.005), implying that CMB is not a serious problem in this research, as shown in Table 4.

**Table 4:** Common method bias.

**Construct**	**Indicator**	**Substantive factor loading (R1)**	**R1^**2**^**	**Method factor loading (R2)**	**R2^**2**^**
Admiration (AD)	AD1	0.724	0.524	0.081	0.007
	AD2	0.859	0.739	−0.052	0.003
	AD3	0.854	0.730	−0.026	0.001
Authenticity (AU)	AU1	0.775	0.600	0.056	0.003
	AU2	0.778	0.605	0.057	0.003
	AU3	0.874	0.763	−0.116	0.013
Consumer engagement (CE)	CE1	0.724	0.525	0.069	0.005
	CE2	0.737	0.543	−0.034	0.001
	CE3	0.719	0.518	−0.005	0.000
	CE4	0.782	0.611	−0.035	0.001
Reputation (RP)	RP1	0.659	0.434	0.047	0.002
	RP2	0.693	0.480	−0.008	0.000
	RP3	0.809	0.655	−0.019	0.000
	RP4	0.496	0.246	0.145	0.021
	RP5	0.792	0.627	−0.118	0.014
	RP6	0.812	0.660	−0.050	0.002
	RP7	0.699	0.488	0.029	0.001
Expertise (EX)	EX1	0.718	0.515	0.003	0.000
	EX2	0.819	0.671	−0.092	0.008
	EX3	0.720	0.518	0.058	0.003
	EX4	0.672	0.451	0.032	0.001
Guarantee (GU)	GU1	0.828	0.686	−0.028	0.001
	GU2	0.773	0.597	0.050	0.003
	GU3	0.844	0.712	−0.022	0.000
Interactivity (IT)	IT1	0.779	0.608	−0.140	0.020
	IT2	0.732	0.535	−0.017	0.000
	IT3	0.624	0.389	0.118	0.014
	IT4	0.655	0.429	0.055	0.003
	IT5	0.761	0.580	−0.028	0.001
Money-saving (MS)	MS1	0.755	0.569	−0.030	0.001
	MS2	0.641	0.411	0.068	0.005
	MS3	0.778	0.605	−0.068	0.005
	MS4	0.752	0.566	0.032	0.001
Psychological distance (PD)	PD1	0.856	0.732	0.117	0.014
	PD2	0.698	0.487	−0.131	0.017
	PD3	0.839	0.704	0.019	0.000
Trust toward live streaming activity (TR)	TR1	0.762	0.581	−0.033	0.001
	TR2	0.697	0.486	−0.019	0.000
	TR3	0.634	0.402	0.116	0.014
	TR4	0.852	0.726	−0.060	0.004
Average		0.749	0.568	0.001	0.005

All indicator loadings are significant (*p* < 0.001).

### Measurement model

We used factor analysis to verify the reliability and validity of the constructs. According to the results, Cronbach's α of all the constructs was between 0.712 and 0.838, higher than 0.7, indicating that the constructs had relatively good reliability. The composite reliability (CR) of each structure was >0.8, which indicates that the internal consistency of the constructs was relatively good. The minimum item loading is 0.707, larger than 0.7, so the internal consistency of the structure was good. The average variance extracted (AVE) of each construct was between 0.500 and 0.664, indicating that these constructs show relatively good convergent validity. The statistical analysis results are listed in Table 5.

**Table 5:** Results of reliability and convergent validity analysis.

**Factor**	**Cronbach's α**	**CR**	**AVE**	**AD**	**AU**	**CE**	**RP**	**EX**	**GU**	**IT**	**MS**	**PD**	**TR**
AD	0.744	0.854	0.662	0.813									
AU	0.734	0.850	0.653	0.323	0.808								
CE	0.724	0.829	0.548	0.544	0.392	0.740							
RP	0.838	0.878	0.509	0.468	0.519	0.505	0.713						
EX	0.712	0.822	0.535	0.315	0.419	0.480	0.374	0.732					
GU	0.747	0.856	0.664	0.370	0.435	0.463	0.383	0.495	0.815				
IT	0.752	0.833	0.500	0.396	0.387	0.475	0.472	0.435	0.457	0.707			
MS	0.712	0.822	0.537	0.371	0.367	0.501	0.347	0.404	0.430	0.478	0.733		
PD	0.712	0.838	0.634	−0.451	−0.400	−0.518	−0.422	−0.343	−0.434	−0.533	−0.410	0.796	
TR	0.721	0.827	0.545	0.464	0.597	0.610	0.575	0.564	0.522	0.539	0.481	−0.475	0.738

AD, admiration; AU, authenticity; CE, consumer engagement; RP, Reputation; EX, expertise; GU, guarantee; IT, interactivity; MS, money-saving; PD, psychological distance; TR, trust toward live streaming activity; CR, composite reliability; AVE, average variance extracted.

We test discriminant validity by three methods (Sun et al., 2019): (1) we compared the relationship between the square root of the AVE of the variable and the correlation coefficients. It can be seen from the analysis results that the square root of the AVE of the variable is greater than the correlation coefficients, see Table 5. Therefore, the constructs also have relatively sound discriminant validity; (2) we utilized cross-loadings to confirm discriminant validity (George, 1998). The results show that all indicator loadings exceed the cross-loadings, verifying discriminant validity, see Table 6; (3) we employed the heterotrait-monotrait (HTMT) ratio to test for discriminant validity. The results show that HTMT values ranged from 0.426 to 0.842, and were lower than 0.85, indicating that the discriminative validity was confirmed (Sun et al., 2019), see Table 7. By utilizing three methods to test discrimination validity, we find that this study satisfies the criteria of discriminant validity.

**Table 6:** Results for discriminant validity.

	**Items**	**VIF**	**Loadings and cross-loadings**									
			**AD**	**AU**	**CE**	**RP**	**EX**	**GU**	**IT**	**MS**	**PD**	**TR**
AD	AD1	1.367	0.792	0.286	0.445	0.383	0.317	0.357	0.349	0.323	−0.393	0.384
	AD2	1.556	0.820	0.248	0.440	0.385	0.231	0.275	0.287	0.272	−0.313	0.370
	AD3	1.604	0.828	0.252	0.441	0.373	0.217	0.267	0.329	0.310	−0.393	0.378
AU	AU1	1.466	0.285	0.814	0.346	0.492	0.355	0.325	0.327	0.310	−0.345	0.488
	AU2	1.477	0.251	0.820	0.342	0.452	0.366	0.386	0.347	0.326	−0.373	0.496
	AU3	1.417	0.248	0.791	0.260	0.308	0.293	0.343	0.261	0.253	−0.246	0.462
CE	CE1	1.446	0.415	0.344	0.786	0.450	0.428	0.427	0.344	0.330	−0.449	0.506
	CE2	1.311	0.335	0.303	0.706	0.388	0.322	0.251	0.302	0.346	−0.332	0.462
	CE3	1.314	0.425	0.207	0.716	0.275	0.310	0.381	0.410	0.440	−0.370	0.412
	CE4	1.397	0.433	0.300	0.749	0.374	0.352	0.299	0.352	0.374	−0.373	0.421
RP	RP1	1.713	0.251	0.424	0.350	0.685	0.287	0.339	0.287	0.338	−0.299	0.413
	RP 2	1.637	0.284	0.355	0.291	0.682	0.288	0.289	0.319	0.222	−0.338	0.407
	RP 3	2.164	0.377	0.439	0.352	0.790	0.267	0.306	0.371	0.227	−0.368	0.448
	RP 4	1.455	0.279	0.268	0.371	0.608	0.285	0.384	0.390	0.282	−0.281	0.354
	RP 5	1.934	0.348	0.367	0.310	0.700	0.220	0.160	0.302	0.202	−0.250	0.342
	RP 6	2.122	0.402	0.380	0.403	0.781	0.238	0.250	0.339	0.218	−0.287	0.444
	RP 7	2.067	0.373	0.354	0.436	0.732	0.296	0.215	0.353	0.263	−0.288	0.450
EX	EX1	1.314	0.167	0.297	0.395	0.279	0.713	0.285	0.303	0.347	−0.240	0.416
	EX2	1.450	0.217	0.289	0.298	0.249	0.737	0.373	0.325	0.201	−0.165	0.390
	EX3	1.406	0.282	0.391	0.348	0.320	0.753	0.421	0.373	0.320	−0.273	0.400
	EX4	1.251	0.246	0.250	0.360	0.246	0.723	0.363	0.275	0.304	−0.307	0.441
GU	GU1	1.477	0.277	0.297	0.401	0.312	0.396	0.806	0.347	0.305	−0.356	0.415
	GU2	1.461	0.308	0.443	0.346	0.353	0.409	0.812	0.388	0.338	−0.368	0.433
	GU3	1.546	0.318	0.320	0.385	0.272	0.404	0.827	0.382	0.408	−0.338	0.429
IT	IT1	1.342	0.165	0.208	0.244	0.290	0.229	0.304	0.642	0.310	−0.304	0.256
	IT2	1.404	0.224	0.287	0.294	0.349	0.269	0.336	0.712	0.367	−0.310	0.421
	IT3	1.367	0.287	0.315	0.382	0.398	0.365	0.346	0.742	0.315	−0.479	0.432
	IT4	1.374	0.429	0.229	0.354	0.326	0.296	0.340	0.688	0.388	−0.382	0.336
	IT5	1.470	0.279	0.310	0.380	0.294	0.354	0.296	0.748	0.323	−0.382	0.425
MS	MS1	1.344	0.273	0.240	0.317	0.232	0.266	0.329	0.372	0.731	−0.273	0.348
	MS2	1.267	0.318	0.268	0.438	0.328	0.352	0.274	0.296	0.686	−0.245	0.327
	MS3	1.348	0.195	0.192	0.340	0.216	0.244	0.323	0.333	0.725	−0.316	0.337
	MS4	1.423	0.301	0.363	0.378	0.246	0.322	0.333	0.393	0.785	−0.359	0.395
PD	PD1	1.361	−0.298	−0.261	−0.373	−0.257	−0.260	−0.303	−0.363	−0.254	0.757	−0.337
	PD2	1.362	−0.396	−0.326	−0.457	−0.429	−0.297	−0.389	−0.457	−0.366	0.809	−0.404
	PD3	1.491	−0.375	−0.361	−0.400	−0.307	−0.259	−0.339	−0.445	−0.349	0.821	−0.389
TR	TR1	1.357	0.371	0.415	0.441	0.420	0.446	0.318	0.363	0.369	−0.369	0.732
	TR2	1.260	0.277	0.443	0.431	0.419	0.389	0.353	0.296	0.303	−0.326	0.684
	TR3	1.349	0.391	0.456	0.431	0.447	0.380	0.420	0.450	0.384	−0.371	0.731
	TR4	1.539	0.332	0.449	0.496	0.414	0.451	0.445	0.472	0.363	−0.339	0.802

AD, admiration; AU, authenticity; CE, consumer engagement; RP, Reputation; EX, expertise; GU, guarantee; IT, interactivity; MS, money-saving; PD, psychological distance; TR, trust toward live streaming activity. For loadings and cross-loadings, the bold values shaded in gray are the indicator loading values corresponding to the constructs, and others are cross-loading values. VIFs, variance inflation factors; all indicator VIFs are below 3, which indicates that there are no major multicollinearity problems. All indicator loadings are significant (*p* < 0.001).

**Table 7:** Heterotrait-monotrait ratio (HTMT).

	**AD**	**AU**	**CE**	**RP**	**EX**	**GU**	**IT**	**MS**	**PD**	**TR**
AD										
AU	0.436									
CE	0.740	0.533								
RP	0.588	0.658	0.644							
EX	0.426	0.578	0.662	0.488						
GU	0.493	0.585	0.624	0.493	0.675					
IT	0.521	0.510	0.634	0.592	0.583	0.610				
MS	0.509	0.501	0.704	0.459	0.563	0.589	0.655			
PD	0.614	0.547	0.714	0.540	0.471	0.591	0.711	0.566		
TR	0.634	0.821	0.842	0.740	0.785	0.710	0.713	0.671	0.662	

AD, admiration; AU, authenticity; CE, consumer engagement; RP, Reputation; EX, expertise; GU, guarantee; IT, interactivity; MS, money-saving; PD, psychological distance; TR, trust toward live streaming activity.

### Structural model

**Figure 3:**
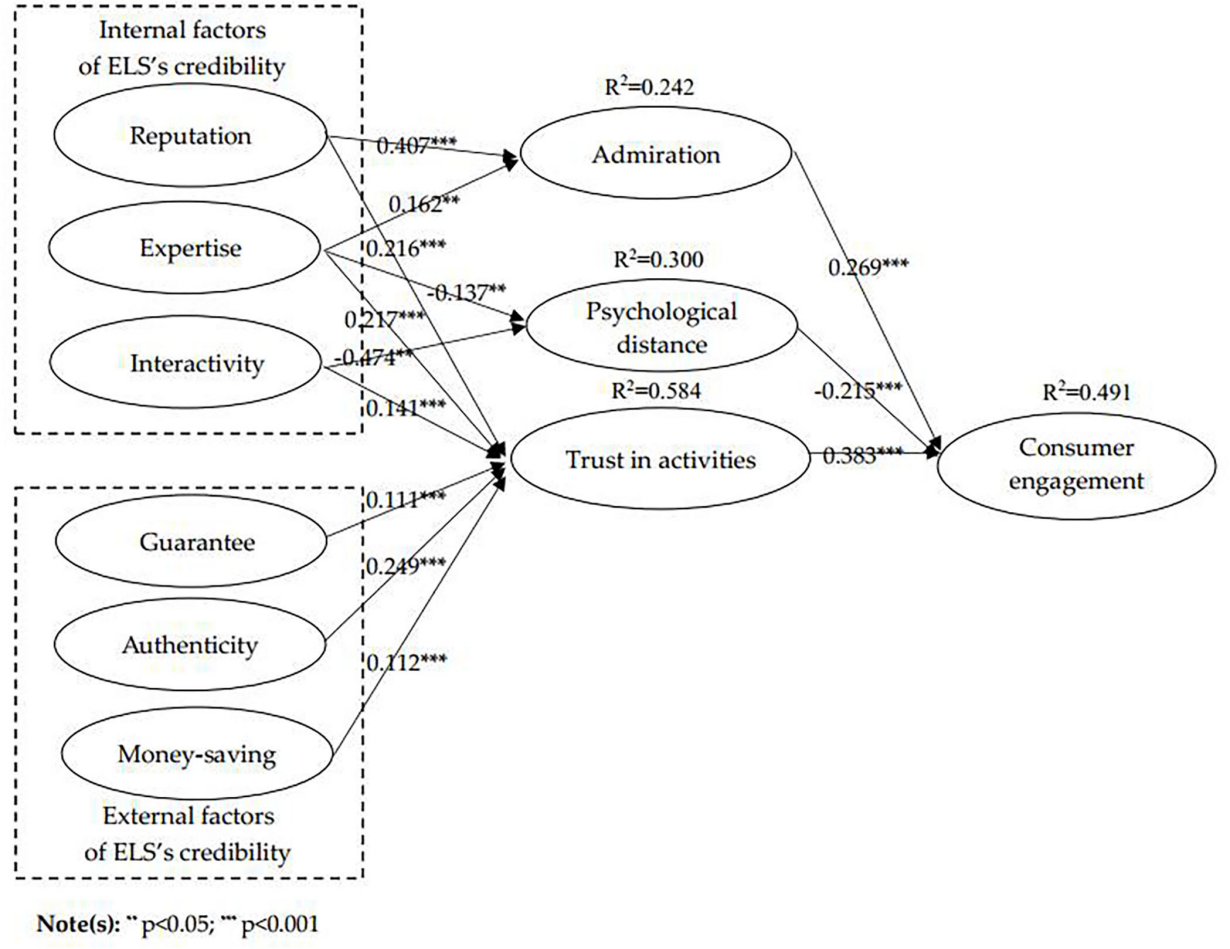
Results of the proposed model.

Figure 3 shows the results of the SmartPLS analysis that reputation (β = 0.216, *p* < 0.001), expertise (β = 0.203, *p* < 0.001), interactivity (β = 0.141, *p* < 0.01), guarantee (β = 0.111, *p* < 0.05), authenticity (β = 0.249, *p* < 0.001), money-saving (β = 0.112, *p* < 0.05) positively affect trust in activities, and trust in activities, positively affects consumer engagement (β = 0.383, *p* < 0.001); reputation (β = 0.407, *p* < 0.001), expertise (β = 0.162, *p* < 0.05) positively affect admiration, and admiration positively affect consumer engagement (β = 0.269, *p* < 0.001); interactivity (β = −0.474, *p* < 0.001) and expertise (β = −0.137, *p* < 0.05) negatively affect psychological distance, and psychological distance negatively affects consumer engagement (β = −0.215, *p* < 0.001). The *R*^2^ for admiration, trust in activities, psychological distance, and consumer engagement is 0.242, 0.584, 0.300, and 0.491, respectively. Thus, H1a–b, H2a–c, H3a–b, H4, H5, H6, H7, H8, and H9 are all supported, as shown in Table 8.

**Table 8:** Results of path analysis.

**Path**	**Coefficient**	***t*-value**	***R* ^2^**	**Hypothesis result**
RP → AD	0.407^***^	6.812	0.242	H1a: supported
EX → AD	0.162^**^	2.177		H2a: supported
EX → PD	−0.137^**^	2.054	0.300	H2b: supported
IT → PD	−0.474^***^	8.194		H3a: supported
RP → TR	0.216^***^	3.715	0.584	H1b: supported
EX → TR	0.217^***^	3.691		H2c: supported
IT → TR	0.141^**^	2.661		H3b: supported
GU → TR	0.111^**^	2.089		H4: supported
AU → TR	0.249^***^	4.652		H5: supported
MS → TR	0.112^**^	2.066		H6: supported
AD → CE	0.269^***^	4.677	0.491	H7: supported
PD → CE	−0.215^***^	3.548		H8: supported
TR → CE	0.383^***^	6.583		H9: supported

** p < 0.05,

*** p < 0.001; AD, admiration; AU, authenticity; CE, consumer engagement; RP, Reputation; EX, expertise; GU, guarantee; IT, interactivity; MS, money-saving; PD, psychological distance; TR, trust toward live streaming activity.

### Mediation analysis

H10a–b posits that admiration mediates the effect of (a) entrepreneur reputation, (b) expertise on consumer engagement; H11a–b posits that psychological distance mediates the effect of (a) expertise and (b) interactivity on consumer engagement; H12a–f posits that trust in activities mediates the effects of (a) reputation, (b) expertise, (c) interactivity, (d) guarantee, (e) authenticity, and (f) money-saving on consumer engagement. A bootstrapping procedure with 5,000 bootstrapped samples was employed to test the mediating effects. If 95% bootstrapped confidence intervals (CI), for the estimates of indirect effect that do not include zero, the mediation effect is statistically significant (Zhao et al., 2010). Table 9 reports the estimates of indirect pathways with their respective 95% CI. The results show that except for H11b and H12f, the indirect effect of H10a–b, H11a, and H12a–e are all significant. Thus, H10a–b, H11a, and H12a–e are supported.

**Table 9:** Mediating effects tests.

**Path**	**Indirect effect**	**95% CI**		**Hypothesis result**
		**Lower**	**Upper**	
RP → AD → CE	0.110^***^	0.055	0.177	H10a supported
EX → AD → CE	0.044^**^	0.004	0.092	H10b supported
IT → PD → CE	0.102^**^	0.045	0.170	H11a supported
EX → PD → CE	0.029	0.001	0.071	H11b not supported
RP → TR → CE	0.083^***^	0.038	0.140	H12a supported
EX → TR → CE	0.083^**^	0.033	0.137	H12b supported
IT → TR → CE	0.054^**^	0.013	0.102	H12c supported
GU → TR → CE	0.042^**^	0.001	0.085	H12d supported
AU → TR → CE	0.095^***^	0.052	0.148	H12e supported
MS → TR → CE	0.043	0.004	0.089	H12f not supported

** p < 0.05;

*** p < 0.001; AD, admiration; AU, authenticity; CE, consumer engagement; RP, Reputation; EX, expertise; GU, guarantee; IT, interactivity; MS, money-saving; PD, psychological distance; TR, trust toward live streaming activity.

## Discussion and conclusion

### Major findings

This research first examined that consumer engagement (i.e., the peak number of viewers, the total amount of viewers, number of viewer's likes, the total amount of tips, and the increased number of fans) of the ELS is significantly higher than that of the employees' live streaming in the enterprise live streaming based on a quasi-experiment. Furthermore, we confirmed the effect of success factors on consumer engagement in the ELS through the online survey. Our findings confirm the effects of internal factors (i.e., reputation, expertise, and interactivity) and external factors (i.e., guarantee, authenticity, and money-saving) of the ELS's credibility on consumer engagement and the mechanisms underlying those effects.

First, we found that factors of ELS's credibility positively affect trust in activities. In terms of internal factors: (1) reputation positively affects trust in activities, which is consistent with the study of Kim and Peterson (2017) illustrated that reputation is an important antecedent of trust. The result reveals that when consumers engage the ELS, they prefer to engage entrepreneur streamers with reputation. (2) Expertise positively affects trust in activities, which is consistent with the study of Guo et al. (2022) suggested that expertise is one of the top streamers' characteristics that can influence consumer behavior. Entrepreneur streamers are the experts on their products that they have rich knowledge about the products. Mingzhu Dong, the chairwoman of the GREE, said that compared with other live streamers, no one is more experiential than her in the air conditioner industry. (3) Interactivity positively affects trust in activities, which is consistent with the study of Arghashi and Yuksel (2022) suggested interactivity has a positive effect on consumer trust. In terms of external factors: (1) guarantee positively affects trust in activities. The finding extends the previous study of Rokonuzzaman et al. (2021) and illustrates that the return policy of retailers can enhance store image and patronage intentions from offline context to live streaming context. In live streaming activity, consumers are worried about after-sales services, thus, the promise and guarantee provided by the entrepreneur streamers is an important prerequisite for consumer trust. (2) Authenticity positively affects trust in activities. In the live streaming activity, consumers worried about false products and misinformation. Authenticity can enhance consumer trust in activities, which is consistent with the study of Matthews et al. (2020). (3) Money-saving positively affects trust in activities. Entrepreneur streamers can offer “entrepreneur price,” the lowest price compared with other promotion channels, which can help gain consumers' trust in activities.

Second, we found the effects of reputation and expertise on admiration. In detail, reputation positively affects admiration, which is consistent with previous studies that the preference originates from an appreciation of excellent behavior and character (Haidt, 2003). Expertise positively affects admiration. This finding is consistent with a previous study that admiration is derived from skills and actions (Algoe and Haidt, 2009). Meanwhile, we found expertise and interactivity negatively affect psychological distance. Expertise negatively affects psychological distance because professional information can reduce information asymmetry (Dimoka et al., 2012). Interactivity negatively affects psychological distance, which is consistent with the study of Xue et al. (2020) for live streaming.

Third, we found the effect of admiration, psychological distance, and trust in activities on consumer engagement. (1) Admiration positively affects consumer engagement and mediates the effect of reputation and expertise on consumer engagement. This finding extends the previous study, showing that consumer admiration for entrepreneurs positively affects brand attitude (Liu et al., 2018). (2) Psychological distance negatively affects consumer engagement, which is consistent with the study of Xue et al. (2020). This point indicates that psychological distance harms social commerce engagement in live streaming. (3) Trust in activities positively affects consumer engagement. This finding is consistent with a previous study, which revealed that trust has a positive relationship with consumer engagement in live streaming (Wongkitrungrueng and Assarut, 2020).

Fourth, this study examined the mediation effect of admiration, psychological distance, and trust in activities on the relationship between the ELS' credibility and consumer engagement. (1) Admiration meditates the effect of reputation and expertise on consumer engagement. The findings show that watching entrepreneur streamers with reputation and skills in live streaming activity can lead to consumer admiration for the entrepreneur and in turn increases consumer engagement. (2) Psychological distance mediates the effect of interactivity on consumer engagement. The finding tells us that consumers feel closer when entrepreneur streamers interact with them and then increase consumer engagement. However, the mediation effect of psychological distance on the relationship between expertise and consumer engagement is not significant. The possible reason is that the psychological distance shortened by expertise came from the decreased information asymmetry (Dimoka et al., 2012), which cannot increase consumer engagement in the ELS. Furthermore, the gender distribution of the sample may be one reason. (3) Trust in activities mediates the effects of reputation, expertise, interactivity, guarantee, and authenticity on consumer engagement. Nevertheless, the effect of money-saving on consumer engagement is not mediated by trust in activities. The likely reason is that money-saving may directly affect consumer engagement.

The findings of this paper provide insights with theoretical and practical implications.

### Theoretical implications

This study provides four implications from a theoretical perspective by investigating the impact of ELS credibility factors on consumer engagement and its underlying mechanisms.

First, we extend source credibility theory in the live streaming literature by providing the credibility of the ELS. Previous live streaming studies based on source credibility mainly were dedicated to the streamers' credibility (Liu et al., 2020; Meng et al., 2020; Park and Lin, 2020). The streamers are one of the important components in live streaming (Guo et al., 2022), but those studies failed to grasp the whole live streaming system. In this study, we extended the study of source credibility in live streaming by studies to examine the factors of ELS's credibility and the effects on consumer engagement based on source credibility theory and signaling theory.

Second, we provided a novel perspective to discuss the ELS-driven consumer engagement from the perspective of internal and external factors of the ELS's credibility. In live streaming, streamers, live streaming platforms, and consumers are important. Previous studies have examined the relationship among streamers, live streaming platforms, and consumers (Wongkitrungrueng and Assarut, 2020; Chen et al., 2021; Guo et al., 2021, 2022), which overlooked another important factor, marketing stimuli in the live streaming. In this study, we extended the live streaming literature by introducing marketing stimuli as external factors of the ELS's credibility.

Third, this study examined the mediating roles of admiration, psychological distance, and trust in activities on the relationship between the factors of the ELS's credibility and consumer engagement. Previous studies have suggested the effect of quickly established social ties and perceived value on consumer engagement (Wongkitrungrueng and Assarut, 2020; Guo et al., 2021). However, few studies have illustrated that admiration, psychological distance, and trust in activities are driven by the ELS's credibility in consumer engagement. This study enriched the live streaming literature on the antecedents of consumer engagement.

Fourth, this study employed a portfolio of methods such as a quasi-experiment and an online survey to confirm our findings. Previous studies on live streaming mainly only used an online survey to examine the effects of factors on purchase intention, consumer engagement, and viewing intention. This study utilized a quasi-experiment to confirm that the effect of the ELS is significantly higher than the employees' live streaming, which brings our study a solid foundation to examine the factors of the ELS's credibility on consumer engagement in the enterprise live streaming context. It is suggested that future studies can take advantage of this approach.

### Practical implications

This study provides a deep understanding of the ELS and can offer actionable guidance to practitioners. Our findings can offer insights not only for the marketers in China where live streaming e-commerce is booming but also for the marketers outside China where live streaming e-commerce is emerging. We highlight the importance of internal factors (i.e., reputation, expertise, and interactivity) and external factors (i.e., guarantee, authenticity, and money-saving) of the ELS' credibility in promoting consumer engagement.

First, based on our findings on the effect of reputation, we suggest that entrepreneurs should pay attention to their reputation in the business field, especially in terms of competency and charisma. With the development of social media, marketing managers can share short clips in live streaming apps before the ELS to show entrepreneurial competence and charismatic reputation-related content. For instance, Mi's official account on Tik Tok provided several interviews of Jun Lei, founder of Xiaomi, for consumers before he participated in live streaming to share his achievement and display his charisma.

Second, based on our findings on the effect of interactivity, we suggest that entrepreneur streamers should pay attention to the interaction with the viewers. Normally, there are other live streamers to help entrepreneur streamers in the ELS, we suggest that other live broadcast streamers should give more time and encourage entrepreneur streamers to interact with consumers. Besides, we suggest that entrepreneur streamers can not only display the features of their product but also illustrate the inner concept of the products and share their entrepreneurial experience, because many consumers who admire the entrepreneur may want to learn about the entrepreneurial experience through the ELS.

Third, based on our findings on the effect of guarantee and authenticity, we suggest that entrepreneur streamers should emphasize the authenticity and guarantee policies of their products. Besides, entrepreneurs can show the manufacturing process of their products through live streaming, which will enhance consumers' trust and engagement. For example, Mingzhu Dong, Chairwoman of Gree, invited online consumers to visit her factories in live streaming activities. However, other types of live streamers cannot ensure the authenticity of the products and provide guarantees to consumers. For instance, 27,270 consumers purchased false bird's nests through a famous internet celebrity live streaming in November 2020. A couple of days later, the news reported that more than 20,000 consumers purchased the low-quality sweater in another famous online celebrity's live streaming activities.

Fourth, based on our findings on the effect of money-saving, it is suggested that entrepreneur streamers should bring various price incentive activities for consumers in the ELS. On the one hand, entrepreneurs can decide the prices of products so that they can give the “entrepreneur price,” which means the lowest price, in the ELS; on the other hand, they can provide some enjoyable promotion activities, such as the special price for certain products/services. For example, Jun Lei, founder of Xiaomi, sold 1,000 pieces of mouse pads for only 1 RMB, which attracted a huge number of consumers.

### Limitations and future research

There are some limitations to this study, which also provide directions for future research. First, although this study has discussed the effect of reputation on consumer engagement, further moderators (e.g., power distance belief) may provide valuable insights for managerial decisions. For instance, power distance beliefs (Jain and Jain, 2018) may influence the effect of reputation on consumer engagement. Besides, some variables, such as interactivity, authenticity, admiration, and trust, are multi-dimensional. Future research can examine the effect of multi-dimensions of those variables on consumer engagement. Second, we meet difficulties in collecting samples. We collaborated with the professional data collection institution to collect valuable samples with the audience's viewing experience in the ELS. Third, this study investigated live streaming engagement through secondary data and questionnaires, future studies can use a field study to measure consumer engagement. Thus, the effects of other interesting factors affecting consumer engagement may be examined. According to Appendix A1 in Supplementary material, there are many industries in which entrepreneur live streaming has been applied. As the industry is very important and can affect consumer engagement. For instance, smart device companies, such as Xiaomi vs. real estate companies, e.g., Kaisa. Future studies can test factors behind consumer engagement from the perspective of an industry. Besides, as there are several live-streaming platforms, future research can compare the differences in consumer attitudes toward the ELS across live-streaming platforms.

## Data availability statement

The original contributions presented in the study are included in the article/Supplementary material, further inquiries can be directed to the corresponding author/s.

## Author contributions

ZJ: conceptualization, methodology, and writing original draft. HW: project administration. HW and ZJ: reviewing and editing. JX: software, validation, funding acquisition, and formal analysis. TZ: visualization, investigation, and data curation. All authors contributed to the article and approved the submitted version.

## Funding

The work described in this paper was supported by funds from the China Postdoctoral Science Foundation (2021M702319), the Ph.D. Start-up Fund of Natural Science Foundation of Guangdong Province of China (No. 2018A030310005), Funds for Sichuan University to Building a World-Class University (No. 20502044C3002), and the National Natural Science Foundation of China (No. 72102238).

## Conflict of interest

The authors declare that the research was conducted in the absence of any commercial or financial relationships that could be construed as a potential conflict of interest.

## Publisher's note

All claims expressed in this article are solely those of the authors and do not necessarily represent those of their affiliated organizations, or those of the publisher, the editors and the reviewers. Any product that may be evaluated in this article, or claim that may be made by its manufacturer, is not guaranteed or endorsed by the publisher.

## References

[B1] Aaker J. L. Garbinsky E. N. Vohs K. D. (2012). Cultivating admiration in brands: warmth, competence, and landing in the “golden quadrant. J. Consum. Psychol. 22, 191–194. 10.1016/j.jcps.2011.11.012

[B2] Addo P. C. Fang J. Asare A. O. Kulbo N. B. (2021). Customer engagement and purchase intention in live-streaming digital marketing platforms. Serv. Ind. J. 2, 767–786. 10.1080/02642069.2021.1905798

[B3] Algoe S. B. Haidt J. (2009). Witnessing excellence in action: the “other-praising” emotions of elevation, gratitude, and admiration. J. Posit. Psychol. 4, 105–127. 10.1080/17439760802650519 19495425 PMC2689844

[B4] Arghashi V. Yuksel C. A. (2022). Interactivity, inspiration, and perceived usefulness! How retailers' AR-apps improve consumer engagement through flow. J. Retail. Consum. Serv. 64, 102756. 10.1016/j.jretconser.2021.102756

[B5] Chan T. K. Cheung C. M. Lee Z. W. (2017). The state of online impulse-buying research: a literature analysis. Inform. Manag. 54, 204–217. 10.1016/j.im.2016.06.001

[B6] Chandon P. Wansink B. Laurent G. (2000). A benefit congruency framework of sales promotion effectiveness. J. Mark. 64, 65–81. 10.1509/jmkg.64.4.65.18071

[B7] Chen H. Zhang S. Shao B. Gao W. Xu Y. (2021). How do interpersonal interaction factors affect buyers' purchase intention in live stream shopping? The mediating effects of swift guanxi. Internet Res. 32, 335–361. 10.1108/INTR-05-2020-0252

[B8] Chen J. Liao J. (2022). Antecedents of viewers' live streaming watching: a perspective of social presence theory. Front. Psychol. 13, 839629. 10.3389/fpsyg.2022.839629 35432106 PMC9008234

[B9] Connelly B. L. Certo S. T. Ireland R. D. Reutzel C. R. (2011). Signaling theory: A review and assessment. J. Manag. 37, 39–67. 10.1177/0149206310388419

[B10] Desmet P. (2014). How retailer money-back guarantees influence consumer preferences for retailer versus national brands. J. Bus. Res. 67, 1971–1978. 10.1016/j.jbusres.2013.11.001

[B11] Dimoka A. Hong Y. Pavlou P. A. (2012). On product uncertainty in online markets: theory and evidence. MIS Q. 36, 395–426. 10.2307/41703461

[B12] Edwards S. M. Jin K. L. Ferle C. L. (2009). Does place matter when shopping online? Perceptions of similarity and familiarity as indicators of psychological distance. J. Interact. Adv. 10, 35–50. 10.1080/15252019.2009.10722161

[B13] Eggers F. O'Dwyer M. Kraus S. Vallaster C. Güldenberg S. (2013). The impact of brand authenticity on brand trust and SME growth: a CEO perspective. J. World Bus. 48, 340–348. 10.1016/j.jwb.2012.07.018

[B14] Ferris G. R. Blass F. R. Douglas C. Kolodinsky R. W. Treadway D. C. (2003). Personal reputations in organizations, in Organizational Behavior: The State of the Science, 2nd Edn, eds Greenberg J. (Mahwah, NJ: Lawrence Erlbaum Associates), 211–246.

[B15] Fornell C. Bookstein F. L. (1982). Two structural equation models: LISREL and PLS applied to consumer exit-voice theory. J. Market. Res. 19, 440–452. 10.1177/002224378201900406

[B16] Friedrich T. Schlauderer S. Overhage S. (2019). The impact of social commerce feature richness on website stickiness through cognitive and affective factors: an experimental study. Electron. Comm. Res. Appl. 36, 100861. 10.1016/j.elerap.2019.100861

[B17] Gao L. Bai X. (2014). Online consumer behaviour and its relationship to website atmospheric induced flow: insights into online travel agencies in China. J. Retail. Consum. Serv. 21, 653–665. 10.1016/j.jretconser.2014.01.001

[B18] Gao X. Xu X. Y. Tayyab S. M. U. Li Q. (2021). How the live streaming commerce viewers process the persuasive message: an ELM perspective and the moderating effect of mindfulness. Electron. Commer. Res. Appl. 49, 101087. 10.1016/j.elerap.2021.101087

[B19] George A. M. (1998). The partial least squares approach to structural equation modeling. Modern Method. Bus. Res. 295, 295–336.

[B20] Grossman S. Stiglitz J. (1980). On the impossibility of informationally efficient markets. Am. Econ. Rev. 70, 393–408. 10.7916/D8765R99

[B21] Guo L. Hu X. Lu J. Ma L. (2021). Effects of customer trust on engagement in live streaming commerce: mediating role of swift guanxi. Internet Res. 31, 1718–1744. 10.1108/INTR-02-2020-0078

[B22] Guo Y. Zhang K. Wang C. (2022). Way to success: understanding top streamer's popularity and influence from the perspective of source characteristics. J. Retail. Consum. Serv. 64, 102786. 10.1016/j.jretconser.2021.102786

[B23] Gupta S. Nawaz N. Alfalah A. A. Naveed R. T. Muneer S. Ahmad N. (2021). The relationship of CSR communication on social media with consumer purchase intention and brand admiration. J. Theor. Appl. Electron. Comm. Res. 16, 1217–1230. 10.3390/jtaer16050068

[B24] Haidt J. (2003). The moral emotions, in Handbook of Affective Sciences, eds Davidson R. J. Scherer K. R. Goldsmith H. H. (Oxford: Oxford University Press), 852–870.

[B25] Hair J. F. Jr. Sarstedt M. Hopkins L. Kuppelwieser V. G. (2014). Partial least squares structural equation modeling (PLS-SEM): an emerging tool in business research. Eur. Bus. Rev. 26, 106–121 10.1108/EBR-10-2013-0128

[B26] Halder D. Pradhan D. Chaudhuri H. R. (2021). Forty-five years of celebrity credibility and endorsement literature: review and learnings. J. Bus. Res. 125, 397–415. 10.1016/j.jbusres.2020.12.031

[B27] Hallanan L. (2019). Amazon Live is Alibaba's Live-Streaming Without the Good Bits. Available online at: https://www.forbes.com/sites/laurenhallanan/2019/03/15/amazon-live-is-alibabas-live-streaming-without-the-good-bits/#34c56a8f94ab (accessed July 15, 2022).

[B28] He H. Chao M. M. Zhu W. (2019). Cause-related marketing and employee engagement: the roles of admiration, implicit morality beliefs, and moral identity. J. Bus. Res. 95, 83–92. 10.1016/j.jbusres.2018.10.013

[B29] Hernández-Ortega B. (2018). Don't believe strangers: online consumer reviews and the role of social psychological distance. Inform. Manag. 55, 31–50. 10.1016/j.im.2017.03.007

[B30] Hollebeek L. D. Glynn M. S. Brodie R. J. (2014). Consumer brand engagement in social media: conceptualization, scale development and validation. J. Interact. Market. 28, 149–165. 10.1016/j.intmar.2013.12.002

[B31] Hou F. Guan Z. Li B. Chong A. Y. L. (2020). Factors influencing people's continuous watching intention and consumption intention in live streaming: evidence from China. Internet Res. 30, 141–163. 10.1108/INTR-04-2018-0177

[B32] Hu M. Chaudhry S. S. (2020). Enhancing consumer engagement in e-commerce live streaming via relational bonds. Internet Res. 30, 1019–1041. 10.1108/INTR-03-2019-0082

[B33] iResearch (2020). The Live streaming E-commerce report in China 2020. Available online at: http://report.iresearch.cn/wx/report.aspx?id=3606 (accessed May 27, 2022).

[B34] Ismagilova E. Slade E. Rana N. P. Dwivedi Y. K. (2020). The effect of characteristics of source credibility on consumer behaviour: a meta-analysis. J. Retail. Consum. Serv. 53, 101736. 10.1016/j.jretconser.2019.01.005

[B35] Jain S. S. Jain S. P. (2018). Power distance belief and preference for transparency. J. Bus. Res. 89, 135–142. 10.1016/j.jbusres.2018.04.016

[B36] Kang J. W. Namkung Y. (2019). The information quality and source credibility matter in customers' evaluation toward food O2O commerce. Int. J. Hosp. Manag. 78, 189–198. 10.1016/j.ijhm.2018.10.011

[B37] Kang K. Lu J. Guo L. Li W. (2021). The dynamic effect of interactivity on customer engagement behavior through tie strength: evidence from live streaming commerce platforms. Int. J. Inf. Manage. 56, 102251. 10.1016/j.ijinfomgt.2020.102251

[B38] Kim J. Lee K. H. (2019a). Influence of integration on interactivity in social media luxury brand communities. J. Bus. Res. 99, 422–429. 10.1016/j.jbusres.2017.10.001

[B39] Kim S. Lee H. (2019b). The effect of CSR fit and CSR authenticity on the brand attitude. Sustainability. 12, 275. 10.3390/su12010275

[B40] Kim S. Park H. (2013). Effects of various characteristics of social commerce (s-commerce) on consumers' trust and trust performance. Int. J. Inf. Manage. 33, 318–332. 10.1016/j.ijinfomgt.2012.11.006

[B41] Kim Y. Peterson R. A. (2017). A meta-analysis of online trust relationships in ecommerce. J. Interact. Market. 38, 44–54. 10.1016/j.intmar.2017.01.001

[B42] Kimiagari S. Malafe N. S. A. (2021). The role of cognitive and affective responses in the relationship between internal and external stimuli on online impulse buying behavior. J. Retail. Consum. Serv. 61, 102567. 10.1016/j.jretconser.2021.102567

[B43] Komiak S. X. Benbasat I. (2004). Understanding customer trust in agent-mediated electronic commerce, web-mediated electronic commerce, and traditional commerce. Inform. Technol. Manage. 5, 181–207. 10.1023/B:ITEM.0000008081.55563.d4

[B44] Ladhari R. Massa E. Skandrani H. (2020). YouTube vloggers' popularity and influence: the roles of homophily, emotional attachment, and expertise. J. Retail. Consum. Serv. 54, 102027. 10.1016/j.jretconser.2019.102027

[B45] Lehman D. W. O'Connor K. Kovács B. Newman G. E. (2019). Authenticity. Acad. Manag. Ann. 13, 1–42. 10.5465/annals.2017.0047

[B46] Li Y. Li X. Cai J. (2021). How attachment affects user stickiness on live streaming platforms: a socio-technical approach perspective. J. Retail. Consum. Serv. 60, 102478. 10.1016/j.jretconser.2021.102478

[B47] Li Y. Peng Y. (2021). What drives gift-giving intention in live streaming? The perspectives of emotional attachment and flow experience. Int. J. Hum.-Comput. Interact. 37, 1–13 10.1080/10447318.2021.1885224

[B48] Liberman N. Trope Y. (2008). The psychology of transcending the here and now. Science. 322, 1201–1205. 10.1126/science.1161958 19023074 PMC2643344

[B49] Lim P. L. Yazdanifard R. (2015). What internal and external factors influence impulsive buying behavior in online shopping? Glob. J. Manag. Bus. Res. 15, 24–32. Available online at: https://globaljournals.org/GJMBR_Volume15/3-What-Internal-and-External.pdf

[B50] Lim S. Cha S. Y. Park C. Lee I. Kim J. (2012). Getting closer and experiencing together: antecedents and consequences of psychological distance in social media-enhanced real-time streaming video. Comput. Human Behav. 28, 1365–1378. 10.1016/j.chb.2012.02.022

[B51] Lin Y. Yao D. Chen X. (2021). Happiness begets money: emotion and engagement in live streaming. J. Market. Res. 58, 417–438. 10.1177/00222437211002477

[B52] Liu F. Meng L. Chen S. Duan S. (2020). The impact of network celebrities' information source characteristics on purchase intention. Chin. J. Manag. 17, 94–104. Available online at: http://manu68.magtech.com.cn/Jwk_glxb/EN/abstract/abstract11697.shtml

[B53] Liu H. Chu H. Huang Q. Chen X. (2016). Enhancing the flow experience of consumers in China through interpersonal interaction in social commerce. Comput. Human Behav. 58, 306–314. 10.1016/j.chb.2016.01.012

[B54] Liu W. Ji S. M. Qi P. H. (2018). Entrepreneur image, consumer-entrepreneur admiration and consumer brand attitude. For. Econ. Manag. 40, 121–136. Available online at: https://qks.sufe.edu.cn/J/WJGL/Article/Details/A0cacad4aa-9883-4d23-82e8-91a7c87189ff

[B55] Liu Y. Li H. Hu F. (2013). Website attributes in urging online impulse purchase: an empirical investigation on consumer perceptions. Decis. Support Syst. 55, 829–837. 10.1016/j.dss.2013.04.001

[B56] Lu S. Yao D. Chen X. Grewal R. (2021). Do larger audiences generate greater revenues under pay what you want? Evidence from a live streaming platform. Market. Sci. 40, 964–984. 10.1287/mksc.2021.1292

[B57] Matthews L. Eilert M. Carlson L. Gentry J. (2020). When and how frontline service employee authenticity influences purchase intentions. J. Bus. Res. 114, 111–123. 10.1016/j.jbusres.2020.04.002

[B58] Mazzarol T. Reboud S. (2020). The entrepreneur, in Entrepreneurship and Innovation. Springer Texts in Business and Economics, eds Mazzarol T. Reboud S. (Singapore: Springer), 35–61. 10.1007/978-981-13-9412-6_2

[B59] Meng L. Liu F. Chen S. Duan S. (2020). Can I evoke you? A study on the influence mechanism of information source characteristics of different types of live broadcasting celebrity on consumers' willingness to purchase. Nankai Bus. Rev. 23, 131–143. Available online at: https://oversea.cnki.net/kcms/detail/detail.aspx?filename=LKGP202001013&dbcode=CJFD&dbname=CJFD2020&v=

[B60] Ohanian R. (1990). Construction and validation of a scale to measure celebrity endorsers' perceived expertise, trustworthiness, and attractiveness. J. Advert. 19, 39–52. 10.1080/00913367.1990.10673191

[B61] Ostrom A. L. Iacobucci D. (2016). The effect of guarantees on consumers' evaluation of services. J Serv. Market. 12, 362–378. 10.1108/08876049810235405

[B62] Park D. J. Berger B. K. (2004). The presentation of CEOs in the press, 1990–2000: increasing salience, positive valence, and a focus on competency and personal dimensions of image. J Pub. Relat. Res. 16, 93–125. 10.1207/s1532754xjprr1601_4

[B63] Park E. J. Kim E. Y. Funches V. M. Foxx W. (2012). Apparel product attributes, web browsing, and e-impulse buying on shopping websites. J. Bus. Res. 65, 1583–1589. 10.1016/j.jbusres.2011.02.043

[B64] Park H. J. Lin L. M. (2020). The effects of match-ups on the consumer attitudes toward internet celebrities and their live streaming contents in the context of product endorsement. J. Retail. Consum. Serv. 52, 101934. 10.1016/j.jretconser.2019.101934

[B65] Podsakoff P. M. MacKenzie S. B. Lee J. Y. Podsakoff N. P. (2003). Common method biases in behavioral research: a critical review of the literature and recommended remedies. J. Appl. Psychol. 88, 879–903. 10.1037/0021-9010.88.5.879 14516251

[B66] Ranft A. L. Zinko R. Ferris G. R. Buckley M. R. (2006). Marketing the image of management: the costs and benefits of CEO reputation. Organ. Dyn. 35, 279–290. 10.1016/j.orgdyn.2006.05.003

[B67] Rokonuzzaman M. Iyer P. Harun A. (2021). Return policy, no joke: an investigation into the impact of a retailer's return policy on consumers' decision making. J. Retail. Consum. Serv. 59, 102346. 10.1016/j.jretconser.2020.102346

[B68] Ruohomaa S. Kutvonen L. (2005). Trust management survey, in International Conference on Trust Management, 77–92. 10.1007/11429760_6

[B69] Schaefer A. D. Pettijohn C. E. (2006). The relevance of authenticity in personal selling: is genuineness an asset or liability? J. Market. Theory Pract. 14, 25–35. 10.2753/MTP1069-6679140102

[B70] Schindler I. Zink V. Windrich J. Menninghaus W. (2013). Admiration and adoration: their different ways of showing and shaping who we are. Cogn. Emot. 27, 85–118. 10.1080/02699931.2012.698253 22780565

[B71] Spence M. (1973). Job market signaling. Q. J. Econom. 87, 355–374. 10.2307/1882010

[B72] Sun W. Gao W. Geng R. (2021). The impact of the interactivity of internet celebrity anchors on consumers' purchase intention. Front. Psychol. 12, 757059. 10.3389/fpsyg.2021.757059 34777160 PMC8578743

[B73] Sun Y. Shao X. Li X. Guo Y. Nie K. (2019). How live streaming influences purchase intentions in social commerce: an IT affordance perspective. Electron. Commer. Res. Appl. 37, 100886. 10.1016/j.elerap.2019.100886

[B74] Tik Tok (2021). Annual Data Report of 2020. Available online at: https://www.sohu.com/a/442893269_441449 (accessed May 27, 2022).

[B75] Trivedi J. Sama R. (2020). The effect of influencer marketing on consumers' brand admiration and online purchase intentions: an emerging market perspective. J. Internet Comm. 19, 103–124. 10.1080/15332861.2019.1700741

[B76] Wagner S. M. Coley L. S. Lindemann E. (2011). Effects of suppliers' reputation on the future of buyer-supplier relationships: the mediating roles of outcome fairness and trust. J. Supply Chain Manag. 47, 29–48. 10.1111/j.1745-493X.2011.03225.x

[B77] Wongkitrungrueng A. Assarut N. (2020). The role of live streaming in building consumer trust and engagement with social commerce sellers. J. Bus. Res. 117, 543–556. 10.1016/j.jbusres.2018.08.032

[B78] Xu P. Fu B. Lyu B. (2022). The influence of streamer's social capital on purchase intention in live streaming e-commerce. Front. Psychol. 12, 748172. 10.3389/fpsyg.2021.748172 35140648 PMC8819172

[B79] Xue J. Liang X. Xie T. Wang H. (2020). See now, act now: how to interact with customers to enhance social commerce engagement? Inform. Manag. 57, 103324. 10.1016/j.im.2020.103324

[B80] Zhao X. Lynch J. G. Jr. Chen Q. (2010). Reconsidering baron and kenny: myths and truths about mediation analysis. J. Consum. Res. 37, 197–206. 10.1086/651257

