# Supplementary material for: Propelling consumer engagement via entrepreneurs' live streaming?
Source: Front Psychol. 2022 Aug 3;13:890707. doi: 10.3389/fpsyg.2022.890707 (PMC9382305; doi:10.3389/fpsyg.2022.890707)
Supplement: Supplementary file 1 [file Data_Sheet_1.docx]

**Appendix**

**Appendix A1. Entrepreneur live streaming statistics**

| Entrepreneur | Enterprise/Position | Start date | Total times | GMV (Million RMB) | TNV (Ten thousand viewers) | Industry |
| --- | --- | --- | --- | --- | --- | --- |
| Linhe Liang | SANY/Chariman | 2020/2 | 3 | 200 | 370 | Truck |
| Jianzhang Liang | Ctrip/ Chariman | 2020/3 | 37 | 200 | 6000 | Tourism |
| Jinbo Qian | RDF/Founder | 2020/3 | 1 | 0.5 | 43.53 | Shoes |
| Gang Chen | Qunar/CEO | 2020/4 | 1 | 16 | 383.5 | Tourism |
| Zhihua Xu | PEAK/CEO | 2020/4 | 1 | 5 | 6.76 | Sportswear |
| Mingzhu Dong | Gree/ Chairwoman | 2020/4 | 13 | 47,600 | 10000 | Appliance |
| Bin Li | NIO/ Founder | 2020/5 | 1 | 128 | 2064 | Electronic Vehicle |
| Yonghao Liu | New Hope Group/ Founder | 2020/5 | 1 | 15 | 7 | Farms |
| Lei Ding | NetEase/CEO | 2020/6 | 2 | 80 | 1600 | Internet |
| Lihua Wei | JUNLEBAO/ President | 2020/6 | 1 | 25 | 1800 | Beverage |
| Jun Liu | Lenovo/ Vice President | 2020/6 | 1 | 100 | 123 | Technology |
| Cheng Wang | TCL/CEO | 2020/6 | 1 | 100 | 254 | Technology |
| Jingbi Li | United Family Healthcare/ Founder | 2020/6 | 1 | 10 | 180 | Healthcare |
| Hailun Chen | HAILUN/ Founder | 2020/8 | 1 | 10 | 18.61 | Instrument |
| Jun Lei | Mi/Founder | 2020/8 | 2 | 398 | 10513 | Technology |
| Zhiguo Wang | SKWORTH/ President | 2020/11 | 1 | 120 | 449.1 | Technology |
| Wei Wang | RUCHEN Group/ Vice President | 2020/11 | 1 | 300 | 300 | Real Estate |
| Xinlan Cheng | Kaisa | 2020/11 | 4 | 6700 | 290 | Real Estate |
| Yin Chen | Huafa/CEO | 2020/11 | 1 | 5.5 | 307 | Real Estate |
| Hui Zhu | YOUWELL/ | 2020/11 | 1 | 3.05 | 71.84 | Real Estate |
| Chuanliang Feng | SNOWSKY SALT Industry Group. LTD/Chairman | 2020/11 | 1 | 8.21 | 58 | Food |
| Weibing Lu | Mi/ Vice President | 2020/12 | 1 | 246 | 3903 | Technology |
| Shaoxiong Zhou | SEPWOLVES/ Chairman | 2021/2 | 1 | 10 | 60 | Clothing |

**Note(s):** GMV: Gross Merchandise Volume; TNV: Total Number of viewers

**Appendix A2. Definition**

| Construct | Definition | Source |
| --- | --- | --- |
| Consumer engagement | Consumer’s behavioral manifestation toward entrepreneur streamer, including purchase behavior and non-transaction behavior (e.g., sharing, like, give tips). | Guo et al. (2021) |
| Reputation | A perceptual identity reflective of the complex combination of the entrepreneur streamer characteristics and accomplishments demonstrated behavior, and intended images presented over a while as observed directly and/or as reported from secondary sources. | Ferris et al. (2003) |
| Expertise | The entrepreneur streamer has knowledge and experience in the products/services, and he/she has the ability and skill to share with viewers in live streaming. | Ohanian (1991); Liu et al. (2020) |
| Interactivity | The degree to which entrepreneur streamers and consumers can act on each other in a real-time way, on the communication medium, and on the messages and the degree to which such influences are synchronized in live broadcasting e-commerce. | Xue et al. (2020) |
| Guarantee | Manufacturers’ guarantee required by law and money-back guarantee provided by entrepreneur streamers. | Desmet (2014) |
| Authenticity | Consumers perceived value of the products, information, and activity provided by the live streaming activity conforms to the information claimed by the entrepreneur streamer. | Lehman et al. (2019) |
| Admiration | Consumers regard the entrepreneur streamers considered praiseworthy or excellent. | Schindler et al. (2013) |
| Psychological distance | The extent to which individuals mentally construe the entrepreneur streamer as being distant from themselves at that moment. | Nira, and Yaacov (2008) |
| Trust in activities | Trust towards the whole live streaming activity, including products, live streamers, and information provided in the entrepreneur live streaming. | Komiak and Benbasat (2004); Kim and Park (2013) |
| Money-saving | The impression of monetary saving is due to the price reductions, special prices, and free samples provided by entrepreneur streamers. | Chandon et al. (2000) |

**Appendix A3. Measurement Scales**

| Variable | Item | Content | Source |
| --- | --- | --- | --- |
| Admiration (AD) | AD1 | I admire the entrepreneur streamer. | Schindler et al. (2013); Algoe and  Haidt (2009) |
|  | AD2 | I am amazed by some achievements of the entrepreneur streamer. |  |
|  | AD3 | I respect the entrepreneur streamer because of his/her achievements. |  |
| Authenticity (AU) | AU1 | The entrepreneur streamer provides authentic products/services for consumers. | Schaefer and Pettijohn (2006); Kim and Lee (2019) |
|  | AU2 | I think the products/services demonstrated by entrepreneur streamer are reliable. |  |
|  | AU3 | The entrepreneur streamer’s promotion activities are genuine. |  |
| Reputation (RP) | RP1 | The entrepreneur streamer is outstanding in his/her field. | Ferris et al. (2003); Park and Berger, (2004) |
|  | RP2 | The entrepreneur streamer is someone with many accomplishments. |  |
|  | RP 3 | The entrepreneur streamer is someone who has advanced performance in his/her field. |  |
|  | RP 4 | The entrepreneur streamer is someone who has a reputation in his/her field. |  |
|  | RP 5 | The entrepreneur streamer is inspiring. |  |
|  | RP 6 | The entrepreneur streamer is confident. |  |
|  | RP 7 | The entrepreneur streamer is charismatic. |  |
| Expertise (EX) | EX1 | The entrepreneur streamer has the product's promotion relevant skills. | Ohanian (1991); Liu et al. (2020) |
|  | EX2 | The entrepreneur streamer has rich experience about the products which he/she recommended. |  |
|  | EX3 | The entrepreneur streamer has professional knowledge about the products. |  |
|  | EX4 | The entrepreneur streamer is skillful in recommending the products. |  |
| Consumer engagement  (CE) | CE1 | I would expect the live streaming activities of the entrepreneur streamer in the App. | Guo et al. (2021); Wongkitrungrueng and Assarut (2020) |
|  | CE2 | I am likely to recommend the entrepreneur live streaming to my friends. |  |
|  | CE3 | I would watch other relevant short videos about the entrepreneur streamers in the App. |  |
|  | CE4 | I would spend my time watching the entrepreneur live streaming. |  |
|  | CE5 | I would purchase products in entrepreneur live streaming. |  |
| Guarantee (GU) | GU1 | The entrepreneur streamer guarantees the quality of products. | Desmet (2014) |
|  | GU2 | The entrepreneur streamer provides a manufacture guarantee by law. |  |
|  | GU3 | The entrepreneur streamer provides a money-back guarantee. |  |
| Interactivity (IT) | IT1 | The entrepreneur streamer is interactive with consumers. | Kim and Lee (2019); Xue et al. (2020) |
|  | IT2 | The entrepreneur steamer can answer the questions and requests of consumers. |  |
|  | IT3 | The entrepreneur streamer can provide relevant information for consumers’ inquiries. |  |
|  | IT4 | The entrepreneur streamer keeps consumers' attention with his/her entrepreneurial experience. |  |
|  | IT5 | The entrepreneur streamer will provide personalized products or information for consumers’ needs. |  |
| Money-saving (MS) | MS1 | The entrepreneur streamer offers discounts to consumers. | Hu and Chaudhry, 2020; Chandon et al. (2000) |
|  | MS2 | The entrepreneur streamer offers discount coupons to encourage purchases. |  |
|  | MS3 | The entrepreneur streamer provides special incentives. |  |
|  | MS4 | Transactions via entrepreneur live streaming activities can receive premiums or special offers. |  |
| Psychological distance (PD) | PD1 | The entrepreneur streamer and consumers are close. | Liberman and Trope (2008); Xue et al. (2020) |
|  | PD2 | The entrepreneur streamer brings consumers’ hearts closer to the commodities/services. |  |
|  | PD3 | The entrepreneur streamer reduces consumers’ strangeness to the commodities/services. |  |
| Trust in activities (TR) | TR1 | The information that the entrepreneur streamer provides through live streaming is believable. | Kim and Park (2013) |
|  | TR2 | The entrepreneur streamer is trustworthy. |  |
|  | TR3 | The entrepreneur streamer realizes his/her promise. |  |
|  | TR4 | The product/service recommended by the entrepreneur streamer is trustworthy. |  |

=
